# Hemocompatibility and cytotoxicity evaluation of additively manufactured and surface-treated 316 L stainless steel aortic stents using laser powder bed fusion (L-PBF)

**DOI:** 10.1007/s10856-026-07073-8

**Published:** 2026-05-27

**Authors:** Philipp Lulla, Lukas Esper, Ulf Noster, Thomas Schratzenstaller, Christof Schmid, Karla Lehle

**Affiliations:** 1 Laboratory for Medical Devices, Faculty of Mechanical Engineering, Technical University of Applied Sciences (OTH) Regensburg, Regensburg, Germany; 2 Regensburg Center of Biomedical Engineering, Facility of University Regensburg and Technical University of Applied Sciences (OTH) Regensburg, Regensburg, Germany; 3 FIT AG, Lupburg, Germany; 4 Material Science and Surface Analytic Lab, OTH Regensburg, Regensburg, Germany; 5 Regensburg Center of Health Sciences and Technology (RCHST), University of Applied Sciences Regensburg, Regensburg, Germany; 6 Department of Cardiothoracic Surgery, University Hospital Regensburg, Regensburg, Germany

## Abstract

New developments are needed in aortic replacement, with current hybrid solutions suffering from insufficient and rigid stent diameters, thus hindering minimization of false lumen in aortic dissection. Laser powder bed fusion (L-PBF) is an attractive method to generate a new-generation aortic stent. This study investigates the effects of 316 L stainless steel samples manufactured using the L-PBF process on the activity of fibroblasts, red blood cells, leukocytes and platelets on the modified surfaces. Cytotoxicity and hemocompatibility were analyzed under static culture conditions using immunofluorescence as well as scanning electron microscopic (SEM) techniques. Surfaces of additively manufactured samples were etched, electropolished, heat‑treated, and mechanically expanded to optimize the material’s mechanical performance. Alone heat treatment increased the ultimate tensile strength from 585 ± 5 MPa to 695 ± 6 MPa. The additive manufactured and post-processed stents were non-cytotoxic (viability, > 70%, independent of the manufacturing status), non-hemolytic (hemolysis rate, < 1%), and were covered with only a few neutrophils (median (IQR), 25 (12-48) per mm^2^) and platelets (cellular coverage, 0.5 - 10%). Material-induced formation of neutrophil extracellular traps (NETs) was low and not quantifiable. More than 80% of adherent platelets presented an activated conformation and increased expression of CD62P. In contrast, neither circulating leukocytes nor platelets in the supernatant showed any material-induced stimulation as detected via flow cytometry. The results described herein are encouraging and suggest that additive manufactured metallic stents are bio- and hemocompatible and an adequate candidate material for personalized stent production in a very short time.

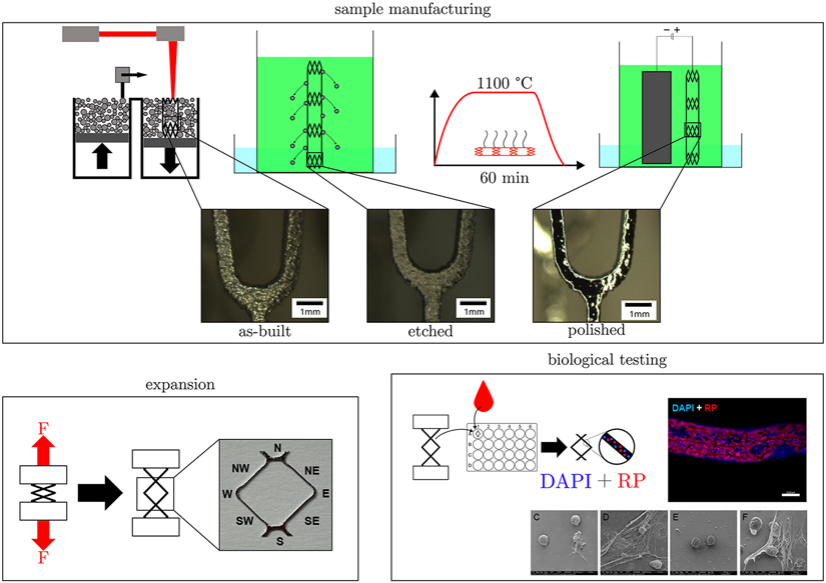

## Introduction

Surgical procedures for life-threatening complex acute aortic dissection involving the aortic arch and the descending thoracic aorta remain challenging. The conventional elephant trunk technique, combined with the thoracic endovascular aortic repair (TEVAR) is an operation with several unresolved issues [1–3]. Hybrid prosthesis associating total arch replacement (TAR) and stent-grafting of the proximal DTA in a single-stage treatment, tremendously facilitate surgery, and promote remodeling of the downstream aorta, also allowing second-stage more distal TEVAR [4–8]. However, the surgical procedure is far from being perfect. In case of an intact dissection membrane within the aortic arch, endovascular stenting would be a much simpler solution than surgical replacement. Moreover, stenting the proximal descending aorta remains unsatisfactory, as the available stents, commonly self-expanding and made of nitinol—the most widely used shape-memory alloy in medical applications—cannot be overexpanded to minimize the false lumen of the aneurysm. Plastically deformable stents made of stainless steel, on the other hand, typically achieve higher radial strength and consequently represent a logical solution. Therefore, new concepts intended to reduce risks and simplify operations are needed.

The additive manufacturing, also called 3D-printing, of stents appears to be an attractive technology to create personalized medical devices. This technology has initialized a medical revolution due to the enormous manufacturing capabilities and the various feeding materials that can be used range from plastics over ceramics to various metallic alloys.

So far, these technologies permit manufacturing of various special prostheses with complex shapes like personalized implants or customized devices in a very short time, especially in the dental, orthopedic and in surgical domains [9–11]. There are also already few approaches that used this technology to create cardiovascular stents of biocompatible steel [12, 13] [12]. realized individualized stents by cast 304 L stainless steel and by Co-Cr alloy with direct metal laser sintering (DMLS), a new additive manufacturing technology [13]. compared 316 L stainless steel stents, manufactured with laser powder bed fusion (L-PBF) and commercial stents. Meanwhile, 316 L is commonly used in medical technology and is widely recognized as a biocompatible material [14–16]. However, adhering particles and rough surfaces are generally considered undesirable for most medical applications, except for orthopedic implants designed to promote bone ingrowth. Surface modifications are therefore necessary to smooth contact surfaces, reducing infection risk [17] and enhancing both mechanical stability and corrosion resistance [18–20]. Although the overall suitability of additively manufactured 316 L as an implant material is given [21], detailed analyses of hemo- and biocompatibility are still lacking. The aim of this study is to investigate the mechanical and biological properties of additively manufactured aortic stents made from 316 L stainless steel. The stents are produced using the Laser Powder Bed Fusion (L-PBF) method, followed by systematic surface modification to enhance mechanical stability and biocompatibility. Detailed analyses of hemocompatibility and cytotoxicity are conducted to evaluate the suitability of these stents for clinical applications and address existing research gaps. The findings aim to provide a deeper understanding of the potential advantages of additively manufactured stents and open new possibilities for personalized medical solutions.

## Materials and methods

### Sample Manufacturing

Tensile specimens (material characterization, mechanical testing) and test samples (surface characterization, biological testing) were built using a L-PBF machine (AMCM M290, AMCM GmbH, Starnberg, Germany) with a maximum power of *P* = 400 W and a spot diameter of about 40 µm within a telecentric optical system.

**Fig. 1:**
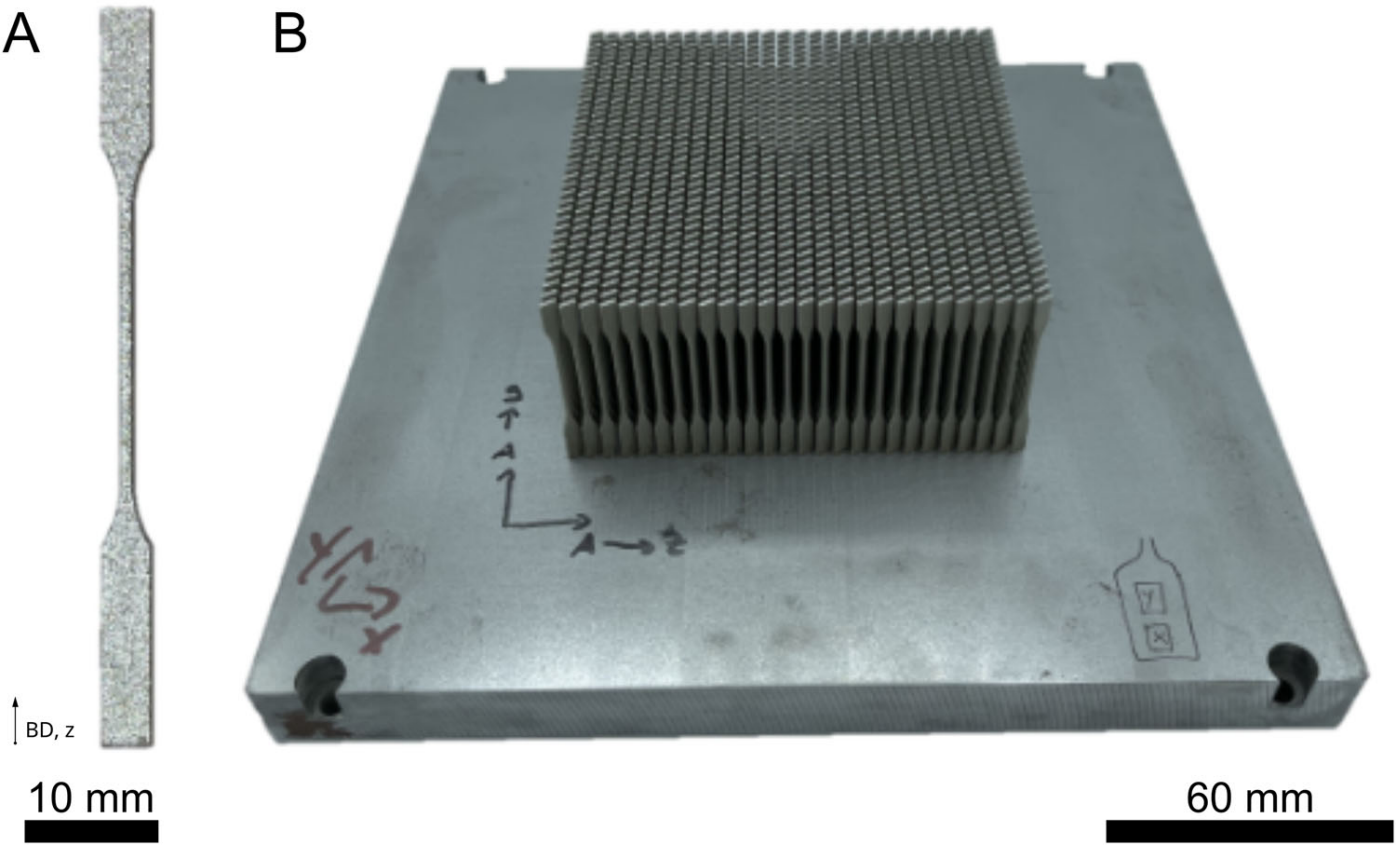
Tensile specimens were built on a batch. **A** Single tensile specimen with a thickness of 1 mm according to DIN 50125, manufactured via L-PBF. **B** Platform with 910 tensile specimens as manufactured after depowdering. BD building direction

The geometry of the tensile specimens met the requirements of the DIN 50125 standard (thickness 1 mm). The specimens were manufactured on a batch with 910 specimens (Fig. 1) to optimize the post-processing of the material used in this study. Printing parameters are listed in Table 1 and provided by the systems manufacturer.

**Table 1:** Printing parameters for tensile specimens

Scantype	Laser power	Scan speed	Layer thickness	Hatch spacing	Offset
	**(W)**	**(mm/s)**	**(µm)**	**(µm)**	**(µm)**
In-fill/hatch	195	1083	20	90	-
Contour	110	800	40	-	0

**Fig. 2:**
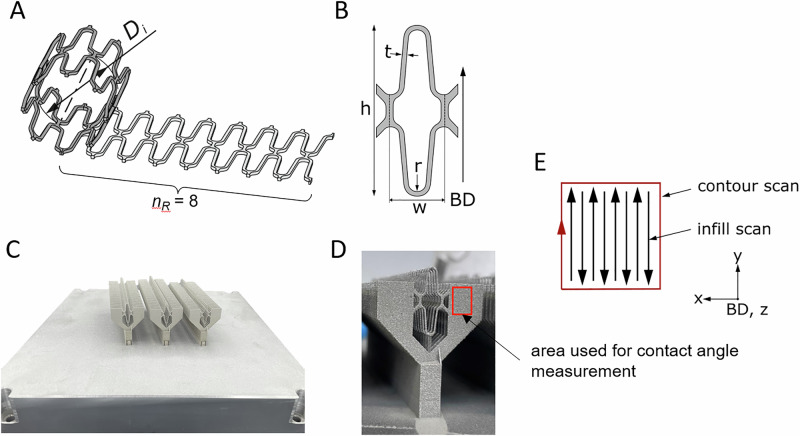
Strategy of test sample manufacturing. **A** Schematic part geometry for a thoracic stent (Di, target diameter of the stent; n_R_, number of rhombi across one D_i_). **B** Scheme of one rhombus of the stent (named, test sample) (h height, w width, t strut width, r strut thickness). Building direction (BD) is indicated by an arrow. **C** Buildjob as taken from the system, in the following called patch with three bars of 40 specimens **D** Manufactured part with area for contact angle measurements (red), 40 samples were placed on one bar. **E** Schematic hatching strategy (with contour scan and infill scan)

The geometry of the test samples was chosen to represent a rhombus of a generic aortic arch stent. To ensure sufficient flexibility with an assumed target diameter of 40 mm (56 mm of the expanded stent), 8 diamonds are assumed across the diameter (Fig. 2A). Thus, leading to a width of w = 6.14 mm and a height of h = 18.7 mm. The strut width is equal to its thickness, t = 0.5 mm. The building direction (BD) is as indicated in the image and resembles a typical building direction for tubular geometries like stents (Fig. 2B). Figure 2D shows a manufactured sample on the build platform of the additive manufacturing system. For easier handling, sheets were printed along the parts. 40 single samples were put on one bar, three bars were manufactured next to each other in one following called patch (Fig. 2C). The samples for this study were taken from the middle bar. For details about the L-PBF process see [22]. Within the L-PBF process gas-atomized 316 L stainless steel powder (Supplementary Table S1) with a particle diameter of 15–45 µm (LPW, Runcorn, United Kingdom) was used.

The test samples were built in 5 independent batches. For the printing process a contour-hatchfill-strategy was used (Fig. 2E). For the hatchfill a hatching pattern with bidirectional scanning was applied. The contour scan was applied with an offset of O = 0 µm. After each layer, the hatching was rotated by an angle of α = 63°. Platform preheating was applied at a temperature of T_substrate_ = 80 °C. Printing parameters used are summarized in Table 2 and were chosen to exceed a density of ${\rho }_{{Printed}}\ge 99.5 \%$ for the given structure.

**Table 2:** Printing parameters for test samples

Scantype	Laser power	Scan speed	Layer thickness	Hatch spacing	Offset
	**(W)**	**(mm/s)**	**(µm)**	**(µm)**	**(µm)**
In-fill/hatch	125	800	40	90	-
Contour	110	800	40	-	0
Support	100	675	40	-	-

### Sample preparation

Both, tensile specimens and test samples were mechanically removed from the building plate to achieve standard stent cell identified as “as-built” condition (AB). To remove lose particles, the samples were cleaned in an ultrasonic bath using ultrapure water (5 min), rinsed in 70% ethanol (70% v/v, prepared from Euro denatured ethanol 99%, VWR Chemicals, Leuven, Belgium), dried and stored in a vacuum desiccator until sample post-processing and sample characterization as well as biological testing.

### Sample post-processing

The post-processing of the samples was carried out according to [22].

1. The samples were chemically etched (CE) for 450 s in a pickling mixture composed of ultrapure water: 37% hydrochloric acid (HCl, 37%, reinst grade, Carl Roth, Karlsruhe, Germany): 65% nitric acid ((HNO₃, 65%, pro analysi grade, Merck, Darmstadt, Germany)) in a ratio of 10:10:1. This removed the powder particles adhering to the surface.
2. The samples were subsequently heat-treated in a vacuum furnace (FRH-100/520/1250 tube furnace, Linn High Therm GmbH, Eschenfelden, Germany) at 1,100 °C for 1 h in order to adjust the microstructure and optimize the mechanical properties (HT, heat-treated).
3. The samples were electrochemically polished (EP) to smooth the surface (Grad et al. 2021). A mineral acid-based electrolyte (Poligrat E268 A, Poligrat Deutschland GmbH, Munich, Germany) at 60 °C was used. Electropolishing was voltage-controlled using a power supply unit (Voltcraft DDPS-32-30, Conrad Electronic SE, Hirschau, Germany) at 3.5 V and 1.1 A for 1200 s.
4. The samples were mechanically elongated to achieve an equivalent diameter of 56 mm, corresponding to the target aortic stent size (ME, mechanically expanded). The elongation process was controlled precisely to 8 mm using a path-controlled universal testing machine. (Hegewald & Peschke inspekt solo 2.5, Hegewald & Peschke Meß- und Prüftechnik GmbH, Nossen, Germany).

All post-processed as well as final test samples (all post-processing steps from AB to ME, termed “final treated” or “TS”) were cleaned in ultrapure water and ethanol and dried until further usage. Samples that were completely post-processed (from AB to ME) were defined as final test sample (termed “final treated” or “TS”).

### Sample characterization

#### Tensile specimens

Material characterization and mechanical testing was described in detail by [23]. Tensile specimens (AB, HT) were used to optimize the quasi-static mechanical properties of the aortic stents using the aforementioned “inspekt solo 2.5” universal testing machine. Tensile tests were performed at room temperature with a constant loading speed (1 mm/min) and a load cell of 2.5 kN. Yield strength (Rp0.2), tensile strength (Rm), uniform elongation (Ag) and elongation at break (A) were calculated.

To investigate the microstructure, tensile specimens (AB, HT) were separated in the measuring range transverse to the building direction and embedded flat in hot mounting resin, ground, polished to a mirror finish, and an electrochemical grain boundary etching was carried out with nitric acid (HNO₃, 65%, pro analysi grade, Merck, Darmstadt, Germany) at 2 V for 10 s at room temperature. The resulting cross sections were analyzed with regard to their grain structure following standard practice [22].

#### Surface roughness of test samples

The surface roughness of the samples (AB, TS) was determined using a LEXT OLS400 laser-scanning microscope (Olympus KK, Tokyo, Japan). A 20x lens and an image section of 648 × 648 μm² was used, observing the surface roughness parameters Ra (mean arithmetic height) and Rz (maximum height). The surface roughness of the implants was measured for three lines per implant (*n* = 9) and the mean value plus standard deviation was calculated.

#### Contact angle measurement

Static contact angle and surface energy were measured by the sessile drop technique using contact angle goniometer (Drop Shape Analyzer DSA25, Krüss, Hamburg, Germany) at ambient conditions. Distilled water (3 µl volume) were used to measure the surface free energy. The measurement was taken immediately after the droplet made contact with the surface to minimize evaporation effects. One measurement was taken on each of five samples (AB, TS) (red-marked area in Fig. 2D), and the surface energies were calculated according to Owens and Wendt [24]. Biomaterials were characterized by critical surface energy of > 55 mJ/m^2^ [25].

### Materials and Methods for biological testing

The methods used for biological testing are known methods to describe the interaction of blood cells with additive manufactured 316 L samples. Details of used antibodies for immunofluorescence (IF) and flow cytometry (FACS), origin of materials, composition of buffers -see Supplementary Material.

#### Test material

Test material consisted of samples in the AB and different post-processing states (see cytotoxicity, Supplementary Material) as well as final test sample (TS) (biological testing) (surface area, 1.04 cm^2^) from 5 different production patches (see 2.2) and thin glass coverslips (CS, round, 1.13 cm^2^, Roth, Karlsruhe, Germany). The test material was incubated in 70% ethanol (30 min, room temperature, RT), dried and transferred into 12-well polystyrene-plates (area, 3.5 cm^2^). Empty wells (well) were used as a reference (no contact with test materials). Just before extract production (cytotoxicity) or cell addition/activation, the wells were moistened with 500 µL sterile PBS, HTP/0.5% BSA or bidestilled water, respectively.

#### Blood donation

The venous blood donations were approved by the local ethics committee of the University of Regensburg (vote: 20-2037-101, date of approval: 2020-10-26) and were taken from healthy volunteers (*n* = 5, free of medications within 10 days before sampling) who had to sign an informed consent form. Blood was collected (21 G butterfly blood collection set) in S-Monovettes with K-EDTA (4 mL) for hemolysis test, lithium-heparin (15 mL) for polymorphonuclear leukocyte (PMN) and peripheral blood mononuclear cell (PBMC) isolation and sodium citrate (3.2%, 17 mL) for preparation of platelet rich plasma (PRP) (Sarstedt, Nümbrecht, Germany).

#### Cytotoxicity evaluation

Cytotoxicity screening (DIN EN ISO 10993-5) ([26] of different test materials to cultured L929 was performed on an extract of the test samples (extraction dilution method) or on the test sample itself (direct contact method).

The L929 mouse fibroblast cell line (NCTC clone 929, ATTC# CCL-1) were cultivated in culture medium (CM, DMEM (Dulbecco´s modified Eagle´s Medium), FBS (fetal bovine serum, 10%), amphotericin/gentamycin, L-glutamine (2 mM), Sigma-Aldrich, Steinheim, Germany) in an incubator (37 °C, 5% CO_2_). Cells were detached with trypsin/EDTA, and seeded (1) into 96-well plates (2,500 cells per well, 150 µL phenol red-free CM, PRF-CM) to be cultivated for 24 h (extraction method), or (2) into 12-well plates (25,000 cells per well, 1 mL CM) to be cultivated for 2 days (direct contact method).

Extraction method: In order to prepare extracts, five samples ( = five different production batches) of each test material as well as copper wire were immersed into PRF-CM in closed vessels and slightly agitated (Platform shaker, Titramax 100, Heidolph, Schwabach, Germany) at 37 °C for 7 days (material surface to extracting vehicle volume ratio, 52 mm^2^/mL). PRF-CM and copper extract (1:6) were used as negative (NC) and positive control (PC). Extracts (150 µL per 96-well) were then added to cultivated L929 for 3 days. Metabolic activity of treated cells was evaluated using the CellTiter 96® AQueous One Solution Cell Proliferation Assay (MTS-test, Promega, Madison, WI, USA) according to manufacturer´s instruction. The absorbance of resulting colour solutions (470 nm/650 nm) was measured (UV–visplate reader, Varioscan Flash, Thermo Fisher Scientific, Waltham, USA) and analyzed using Gen5 software. Results were determined relative to the NC (%). The morphology of extract treated cells (monolayer or spherical detached cells) was documented using an inverted fluorescence phase-contrast microscope (Keyence BZ-8100E, Neuisenburg, Germany) at 16x magnification.

Direct contact method: L929 were seeded into 12-well plates equipped with CS or TS and cultivated for 2 days. Adherent cells were fixed with 1% paraformaldehyde (30 min, RT), washed with PBS, and stained with Rhodamine-Phalloidin (RP, 33 nM in PBS, 500 µL per well, 1 h, RT, darkness). After washing with PBS, CS and TS were transferred to microscopic slides and embedded with FluoromountG-DAPI (Invitrogen, Carlsbad, CA, USA). Twenty to twenty-five photographs from the red channel (RP, 540 nm/565 nm) and the blue channel (DAPI, 358 nm/461 nm) (excitation/emission wavelength) from randomly selected locations along the surface samples (400x magnification) documented the cellular surface coverage. Cell adhesion (number of DAPI-stained nuclei per mm^2^) was quantified using the Keyence microscope.

#### Hemolysis assay

Hemolysis assay was performed according to [27, 28]. Freshly collected EDTA blood was diluted with physiological sodium chloride (NaCl, 0.9%) (4:5). One mL was transferred per 12-well (well, CS, TS) and incubated at 37 °C for 1 h. Negative (NC) and positive controls (PC) were provided by mixing blood with NaCl and distilled water (4:5), respectively. After incubation, the solution of each well was collected and centrifuged (1,200*x* g, 10 min). The amount of hemoglobin released from lysed red blood cells (RBC) was quantified by spectrophotometry at 540 nm. Hemolysis rate was calculated relative to the difference of the absorption of the PC and NC.

#### Platelet adhesion and activation

This analysis was performed with the use of platelet-rich-plasma (PRP). Citrate-anticoagulated blood remained untreated for 30 min (slight movement) and centrifuged (300 x g, 10 min, RT). PRP (700 µL) were removed from the supernatant. The remaining PRPs were centrifuged three times (3320 x g, 10 min, RT) to prepare PPP (platelet poor plasma). PPPs were pooled and used as a diluent for the PRP (700 µL PRP + 6.3 mL PPP; PRP (1:10)).

Each 12-well with CSs and TSs (non-coated) or empty well was filled with 500 µL PRP (1:10) for 1 h at 37 °C. Platelets in the supernatant (non-adherent platelets) were collected to verify material induced platelet activation (fibrinogen-binding and surface expression of CD61, CD62P, and PAC1) using flow cytometry (FACS). Adherent platelets on top of surface samples were washed carefully with PBS and fixed with 1% paraformaldehyde (30 min, RT) (RP, IF) or 2.5% glutaraldehyde (Merck, diluted in PBS) (1 h, RT) (scanning electron microscopy, SEM).

Remaining PRPs in a separate tube were used as non-treated controls (without surface contact) to determine the activation status (FACS) of the platelets at the time of isolation and after 1 h incubation time. In addition, these cells were stimulated with adenosine diphosphate (ADP, 50 µM) or remained non-stimulated (4 min, 37 °C) to differentiate between activated and non-activated cells.

#### PMN and PBMC adhesion and activation

This analysis was performed with the use of isolated leukocytes (PMN, PBMC). The cells were separated using density gradient centrifugation according to [29]. Briefly, equal volumes of LeukoSpin® medium, PBMC Spin® Medium (both pluriSelect Life Science, Leipzig, Germany) and lithium-heparinized whole blood were carefully stacked in a 15 mL tube. After centrifugation (1000 x g, 45 min, RT, without braking), the PBMC layer and the PMN layer (800–1000 µL) were transferred into new 15 mL tubes containing each 2 mL HTP/0.5%BSA. Tubes were centrifuged (250 x g, 10 min, RT) and resuspended in 4 mL HTP/0.5%BSA. On average, 0.7 to 1.5 × 10^7^ PMNs (1.3 to 2.1 × 10^7^ PBMCs) were available for the experiments.

##### PMN adhesion and NET formation

CS coated with Poly-L-Lysine (PLL) were used to demonstrate the adhesion of isolated PMNs and the formation of NETs after stimulation with PMA (Phorbol Myristate Acetate). PLL-coating: CSs were treated with hydrochloric acid/ethanol (1%/70%) (30 min, RT), washed with H_2_O (6x, 5 min, RT), coated with PLL (0.01%, 5 min, RT), and dried until usage. PLL-coated CSs and non-coated TSs were transferred into 12-well plates, moistened with HTP/0.5%BSA and colonized with 1.0 × 10^6^ PMNs per well (500 µL, 30 min, 37 °C). PMN adhesion and material-induced NET-formation on TS was detected after additional 3 h in an incubator. PMNs that adhered onto PLL-coated CSs were stimulated with PMA (100 nM) or remained unchanged (non-stimulated) for 3 h. At the end of the adhesion experiment, non-adherent PMNs in the supernatants (empty well, CS, TS) were collected to determine material-induced cell activation (surface expression of CD11b and CD62L) using FACS. Adherent PMNs were washed with HTP/0.5%BSA and fixed with 1% paraformaldehyde (30 min, RT) or fixed with 2.5% glutaraldehyde (1 h, 4 °C) and used for IF and SEM analysis, respectively. As a positive (negative) control, remaining PMNs were used to demonstrate their activation status (FACS). PMNs were stimulated with tumor necrosis factor (TNF, 10 ng/mL, Thermo Fisher Scientific) and N-formylmethionyl-leucyl-phenylalanine (fMLP, 100 nM, Sigma-Aldrich) (30 min, 37 °C) (activated) or remained non-activated.

##### PBMC adhesion and activation

PLL-coated CSs were used to demonstrate the adhesion of isolated PBMCs and their activation after stimulation with TNF/fMLP (see PMNs). TS were coated with AB-serum (30% in HTP, 16 h, RT). PLL-coated CSs, serum-coated TSs and empty wells were colonized with 2.0 × 10^6^ PMNs per well (500 µL, 3 h, 37 °C). Non-adherent PMNs in the supernatants (empty well, CS, TS) were collected to determine material-induced cell activation (surface expression of CD45, CD14, CD11b and CD62L) using FACS. Adherent PBMCs were treated as PMNs for IF and SEM analysis. As a positive (negative) control, isolated PBMCs were stimulated with TNF/fMLP (activated) or remained non-activated (30 min, 37 °C) as described for PMNs.

#### **Methods to visualize cell adhesion and activation status**

##### Rhodamin-Phalloidin staining (RP) of adherent platelets

TSs and CSs with adherent platelets were washed with PBS/0.5% BSA and stained with RP (33 nM, 1 h, RT). After washing with PBS, the surface samples were mounted with Fluoromount® to visualize RP-stained platelets with the Keyence microscope (red channel, 16x magnification). Twenty randomly selected locations along the TS were photographed. The total density of RP-stained cells per image (platelet adhesion area) was calculated using ImageJ (supplementary material). The calculation also included the total amount of particles per mm^2^, which was differentiated according to area extent ( < 10, 10–100, > 100 mm^2^) to demonstrate single platelets, small and large platelet aggregates.

##### Immunofluorescence staining (IF) of adherent platelets, PMNs and PBMCs:

TSs and CSs with adherent platelets were washed with TBS (3 × 5 min, RT) and TBST0.1% (1 × 10 min, RT). The samples were blocked with blocking buffer (2 h, RT) and incubated with the primary antibodies (rabbit anti-human CD61, mouse anti-human CD62P, each 1:200 diluted with blocking buffer) (4 °C, 16 h). Samples were washed consecutively (each 5 min, RT) 1x TBST0.05%, 1x TBST0.005% and 4x TBS and incubated with secondary antibodies (anti-rabbit-AF488, anti-mouse-AF594, each 1:300 diluted with TBS) (1 h, RT, darkness). After washing with TBS (4x, 5 min, RT), the surface samples were mounted with FluoromountG® (Invitrogen). As positive (negative) controls, adherent platelets on additional CSs were stimulated with ADP (50 µM, 4 min) (or PBS/0.5%BSA), washed, fixed and stained as described above. Stained cells were visualized using Keyence microscope (1600x magnification; green channel, CD61-FITC; red channel, CD62P-PE). The CD61-staining allowed a differentiation in small and plane platelet shapes. The CD62P-staining visualized stimulated cells (punctuated coloring). The overlay identified different platelet populations (small platelets: CD61 + /CD62P + ; or CD61 + /CD62P-; plane platelets CD61 + /CD62P+ or CD61 + / CD62P-).

Adherent PMNs were washed with TBS (3 × 5 min, RT) and stained with SytoxGreen (350 µL, 30 min, RT). According to platelets, samples were washed, blocked and incubated with the primary antibody (rabbit anti-human MPO, 1:1200) (16 h, 4 °C). Samples were washed and incubated with a secondary antibody (anti-rabbit- AF594, 1:300 diluted with TBS) (1 h, RT). Surface samples were mounted with FluoromountG® + DAPI (Invitrogen). Additional CS (non-activated) and CS (PMA) were used as controls to visualize typical PMNs and NET formation (TNF/fMLP and PMA). Stained cells were visualized using Keyence microscope (16x magnification; blue channel, DAPI; green channel, SG; red channel, MPO-AF594). Images (27 ± 9 per TS) of the overlayed channels were used to calculate PMN density (DAPI-stained nuclei per mm^2^, MPO + /DAPI+ cells per mm^2^) and the proportion of NET structures (negative, less (+), moderate (++), extended (+++)) per total amount of PMNs.

Adherent PBMCs were stained with primary antibodies (mouse anti-human CD11b, 1:200; rabbit anti-human CD62L, 1:100) and were visualized with secondary antibodies (donkey anti-rabbit IgG-AF594, donkey anti mouse-AF488, both 1:300). Stained surface samples were embedded in FluoromountG® + DAPI and visualized with Keyence microscope (200x magnification, blue channel, DAPI; green channel, CD11b-AF488; red channel, CD62L-AF594). Images (14–38 per TS) of the overlayed channels were used to calculate PBMC density. Non-stimulated PBMCs were CD11b-low/CD62L + , while stimulated PBMCs were CD11b-high/CD62L- ([30].

Due to non-planar surface geometry, the edge regions of the test samples (that were stained with fluorophore-conjugated antibodies) appeared blurred. The top level of the test samples was focused and only the area with sharply defined cells was evaluated.

##### Scanning electron microscopy (SEM):

Fixed samples (specimens) were dehydrated using differential concentrations of highly pure ethanol (30, 50, 70, 90, 96, and 100%; ROTH, Germany) followed by critical point drying. After drying, the specimens were mounted on aluminum stubs (12.5 mm Ø BP 2152, Baltic Präparation, e.K., Wetter, Germany) using double-sided adhesive carbon discs and conductive adhesive paste (Leit-Tabs 12 mm Ø G 3347, Baltic Präparation, e.K). Then, the specimens were sputtered (BAL-TEC SCD 005, Balzers, Liechtenstein) with a platinum layer thickness of approximately 5 nm (platinum foil BP 2228, Baltic Präparation, e.K). The specimens were subsequently examined in a scanning electron microscope (FEI Quanta 400 FEG, FEI Company, ThermoFisher Scientific, FEI Deutschland GmbH, Frankfurt/Main, Germany; high vacuum mode, secondary electron mode, accelerating voltage 4 kV, spotsize 3, working distance 6–8 mm, aperture 30 µm). The morphologies of adhered platelets were characterized by and classified according to platelet shape [31, 32] (1, round or discoid, no pseudopodia present; 2, dendritic, early pseudopodial, no flattening; 3, spread dendritic, more pseudopodia flattened, partial hyaloplasm between pseudopodia; 4, fully spread, hyaloplasm fully spread, no distinct pseudopodia, formation of collagen networks) [33]. Surface samples (CS, TS) with adherent PMNs and PBMCs were treated accordingly.

#### Flow cytometric analysis of cells in the supernatant after contact with additive manufactured stents

For fibrinogen-binding, platelets (100 µL) were incubated with Fibrinogen-AF488 (1.5 µL, 4 min, RT), fixed with 1% paraformaldehyde (100 µL, 30 min, RT), washed with PBS/0.5%BSA (2 mL), and centrifuged (520x g, 10 min, RT). The cells were stained with CD61-PerCPCy5.5 antibody (5 µL, 45 min, RT), washed, centrifuged and resuspended in 500 µL PBS/0.5%BSA. Another aliquot of the platelets (100 µL) was stained with PAC1-FITC and CD62P-PE antibodies (each 5 µL, 45 min, RT), fixed, washed, and resuspended in 500 µL PBS/0.5%BSA. Isolated platelets were stimulated with 50 µM ADP or non-stimulated (4 min, 37 °C) to differentiate between activated and non-activated cells.

Non-adherent PMNs and PBMCs from the supernatants (500 µL) were centrifuged (250 x g, 10 min). PMNs were stained with anti-human CD62L-FITC and CD11b-PE antibodies. PBMCs were stained with anti-human CD14-FITC, CD45-APC, CD11b-PE and CD62L-PerCPCy5 antibodies (45 min, RT). Cells were fixed, washed, and resuspended in PBS/0.5%BSA. Isolated PMNs or PBMCs immediately after isolation as well as after 3 h in tubes were treated (buffer or TNF/fMLP) and stained with the antibodies in the same way to differentiate between activated and non-activated cells.

A FACS Calibur™ flow cytometer (BD corporate, Franklin Lakes, NJ, USA) and CellQuest Pro software™ (version 5.2, BD corporate, Franklin Lakes, NJ, USA) was used. Platelets were identified by CD61-positive fluorescence and 90 degrees light scatter (SSC) (Supplementary Fig. S1, D). This population was subdivided into non-stimulated platelets (Fibrinogen-FITC-negative) or stimulated platelets (Fibrinogen-FITC-positive). These platelet populations were measured without and with prior activation with ADP. The other aliquot identified platelets according to their forward (FSC) and side scatter (SSC) signal. These cells were divided into PAC1-FITC-positive and/or CD62P-PE-positive (or both) platelet populations. The proportion of positive cells as well as its median fluorescence intensity (median FI) was analysed (Supplementary Fig. S1A, B). PMNs were identified by their typical forward-scatter and side-scatter light patterns and presented as a dotplot (CD11b-PE vs CD62L-FITC). Isolated PMNs were identified with a positive signal for CD62L and a low signal for CD11b. After activation with TNF/fMLP, the majority of PMNs expressed a higher signal for CD11b while only 10% of PMNs lost CD62L. PBMCs were subdivided according to the CD45 and CD14 signals. Monocytes were CD45 + /CD14 + . A further splitting of the monocytes resulted in a high CD11b-PE signal and a high CD62L-PerCPCy5.5 signal (CD11b + /CD62L + ). Already the freshly isolated PBMCs were activated (with high CD11b signal). After 3 h in a tube, the gated monocytes lost CD62L signal as a second activation marker.

### Statistics

SigmaStat 3.5 (SYSTAT Software, San Jose, CA, USA) was used for statistical analysis. Continuous variables were shown as median (interquartile range, IQR), categorical variables are expressed as frequencies (percentage). One-way analysis of variance (ANOVA) was used to compare the parameters as indicated. The Chi-square test was used if nominal distributed parameters were to be tested for correlation. A *p*-value < 0.05 was considered the threshold of statistical significance.

## Results

### Influence of the heat treatment on the microstructure

**Fig. 3:**
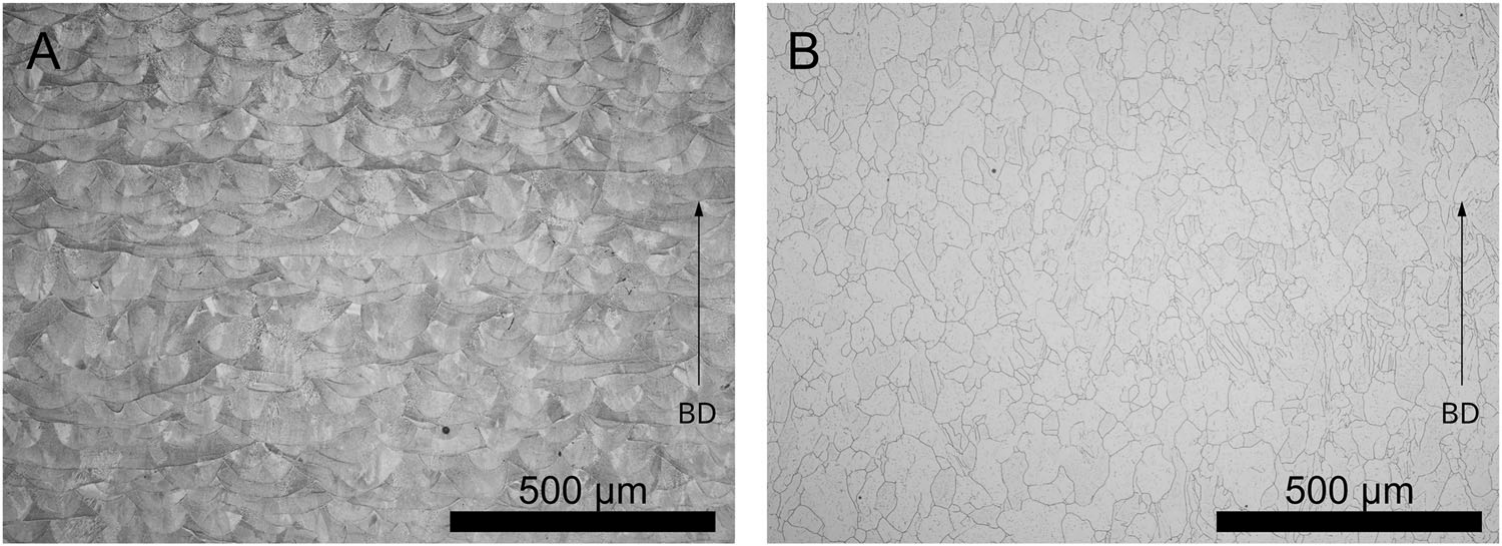
Microstructure of the additively manufactured stent material in the as-built state (AB, **A**) and in the heat treated state (HT, **B**)

The purpose of the post-treatment is to adapt the mechanical properties of the stent material to the application and to reduce the surface roughness. The mechanical properties of the AB and HT materials are shown in Table 3. By changing the microstructure through heat treatment, the yield strength significantly decreased from 484 ± 7 MPa to 413 ± 4 MPa (p < 0.01), the tensile strength significantly increased from 585 ± 5 MPa to 695 ± 6 MPa (*p* < 0.01), and the formability remained unchanged. The microstructure of the tensile specimens in the AB state showed elongated grains and melt pools in the entire imaging area (Fig. 3). In addition, small honeycomb-shaped structures were found within the structures, which are shown and described in [34]. Heat treatment changed the microstructure (Fig. 3B) presenting nearly uniform grains without melt pools [34].

**Table 3:** Mechanical properties of the tensile specimen in the as-built state (AB) and in the heat-treated state (HT).

	Rp0.2**	Rm**	Ag	A
	**(MPa)**	**(MPa)**		
AB	484 ± 7	585 ± 5	0.37 ± 0.02	0.42 ± 0.03
HT	413 ± 4	695 ± 6	0.39 ± 0.00	0.45 ± 0.02

Comparison of the yield strength (Rp0.2), tensile strength (Rm), uniform elongation (Ag) and elongation at break (A) (*n* = 5) **indicates *p* < 0.01

### Post-Treatment Enhancements for Implant Surfaces

**Fig. 4:**
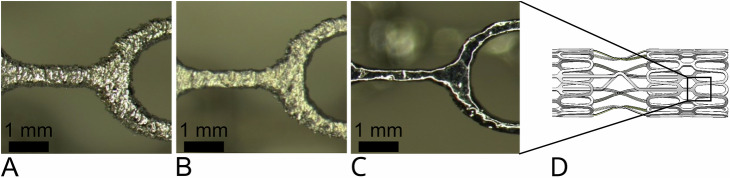
Effect of post-processing on the surface roughness. Surface post-treatment of the stent struts from (**A**): as-built (AB) condition to (**B**): chemically etched (CE) and (**C**): final post-treatment (final treated state, TS) on (**D**): a schematic CAD drawing of an aortic stent specimen

The material in the AB state has a rough surface with particles adhering to the surface (Fig. 4A, B) with a surface roughness Rz of 15.32 ± 2.73 µm and Ra of 2.90 ± 0.60 µm (Table 4). For the post-treatment step explained above, these particles must be removed from the surface, as they would otherwise sinter more strongly. Therefore, the samples (struts) are first etched until the surface is particle-free (Fig. 4CE). The surface is then post-treated using electropolishing to obtain a smooth and reflective surface (Fig. 4TS). A significant reduction in the Rz and Ra value was achieved through the post-treatment (Table 4).

**Table 4:** Surface roughness of the stent struts in the as-built (AB) and final treated state (TS).

	Rz**	Ra**
	**(µm)**	**(µm)**
AB	15.32 ± 2.73	2.90 ± 0.60
Final treated	2.76 ± 2.05	0.44 ± 0.23

Rz (average distance between the highest peak and lowest valley) and Ra (average of profile height deviations from the mean line), each measured cross the building direction (*n* = 9) ** indicates *p* < 0.01

Water contact angles of TS samples (46° ± 2°) are significantly lowered after complete post-treatment of AB samples (57° ± 5°, *p* < 0.01). The observed increase in contact angle of water indicates significant increment of surface energy (AB, 55 mJ/m^2^; TS, 61 mJ/m^2^). However, the surface energy of both AB and TS was above the critical surface energy for biomaterials ( > 55 mJ/m^2^) [25] that should enable cell adhesion and spreading.

### **Cytotoxicity of additive manufactured stents**

**Fig. 5:**
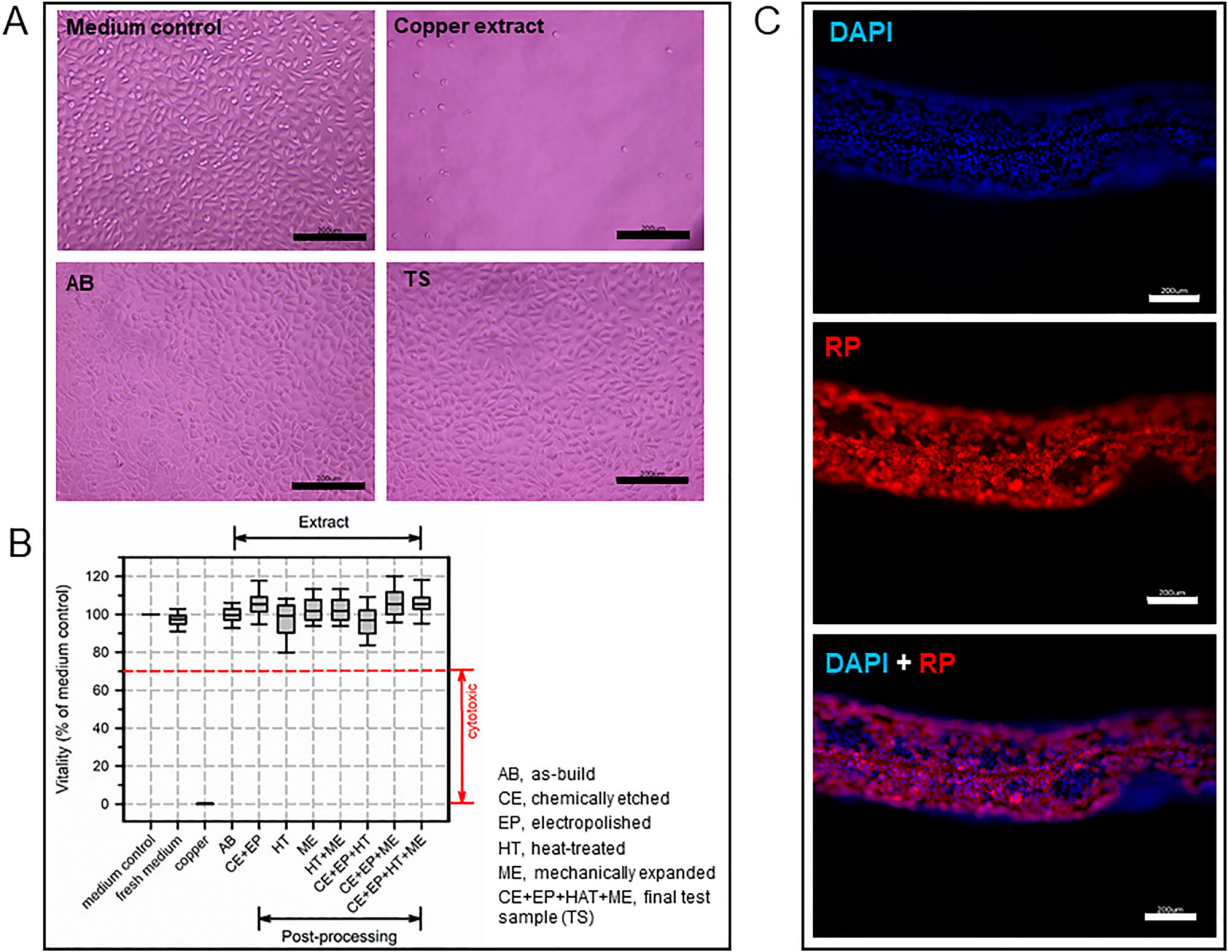
Cytotoxicity of additive manufactured samples. Extracts from samples with different surface treatments were prepared (7 d, 37 °C), cocultured with proliferating L929 fibroblasts for 3 days and visualized with a phase-contrast microscope (scale bar, 200 µm) (**A**). Vitality of treated cells was determined using the MTS-assay relative to the medium control. Reference for non-cytotoxicity was > 70% (red dashed line) (**B**). Attachment of L929 onto the final test sample (TS) visualized by DAPI and rhodamine-phalloidin (RP) stainings using a fluorescence microscope (*n* = 5) (scale bar, 200 µm). Due to non-planar surface geometry, the edge regions of the samples appear blurred (**C**). Boxplots are median and IQR

Different post-processing strategies were examined for their cytotoxic effects using the indirect extraction testing according to ISO 10993-5 (Fig. 5B). Extracts from copper wires contained cytotoxic copper ions that induce cell death in L929 fibroblasts in contrast to medium controls (Fig. 5A). In contrast, extracts from all samples without (AB) and with different post-processings (Fig. 5B) showed no cytotoxic effect L929. None of the five different production batches of the AB as well as the TS presented cytotoxic effects (not shown).

The direct contact of L929 fibroblasts with the surface of the final TS resulted in a complete cellularization as verified by nuclear (DAPI) and cytoskeleton staining (RP, rhodamine-phalloidin) (Fig. 5C). Median (IQR) cell density on TS samples was 1.480 (784–2838) cells per mm^2^.

### Compatibility under isolated blood cells

RBCs, leukocytes and platelets are the major components of human blood and responsible for inflammatory and coagulation processes in response to artificial surfaces such as vascular stents. Additive manufactured metallic stents (TS) were incubated with isolated blood cells and their response was determined under static conditions (both adherent cells and cells in the supernatant).

#### Hemolysis of RBCs after contact with additive manufactured metal stents

Hemolysis measures the amount of free hemoglobin after incubation of surface samples with diluted blood. The hemolysis rate was comparable for the surface samples and the negative control (without surface contact) ( < 1%) and was below 5%, meeting the national biology material hemolysis rate security specified requirements [35, 36].

#### Immunological response of PBMCs with additive manufactured metal stents

**Fig. 6:**
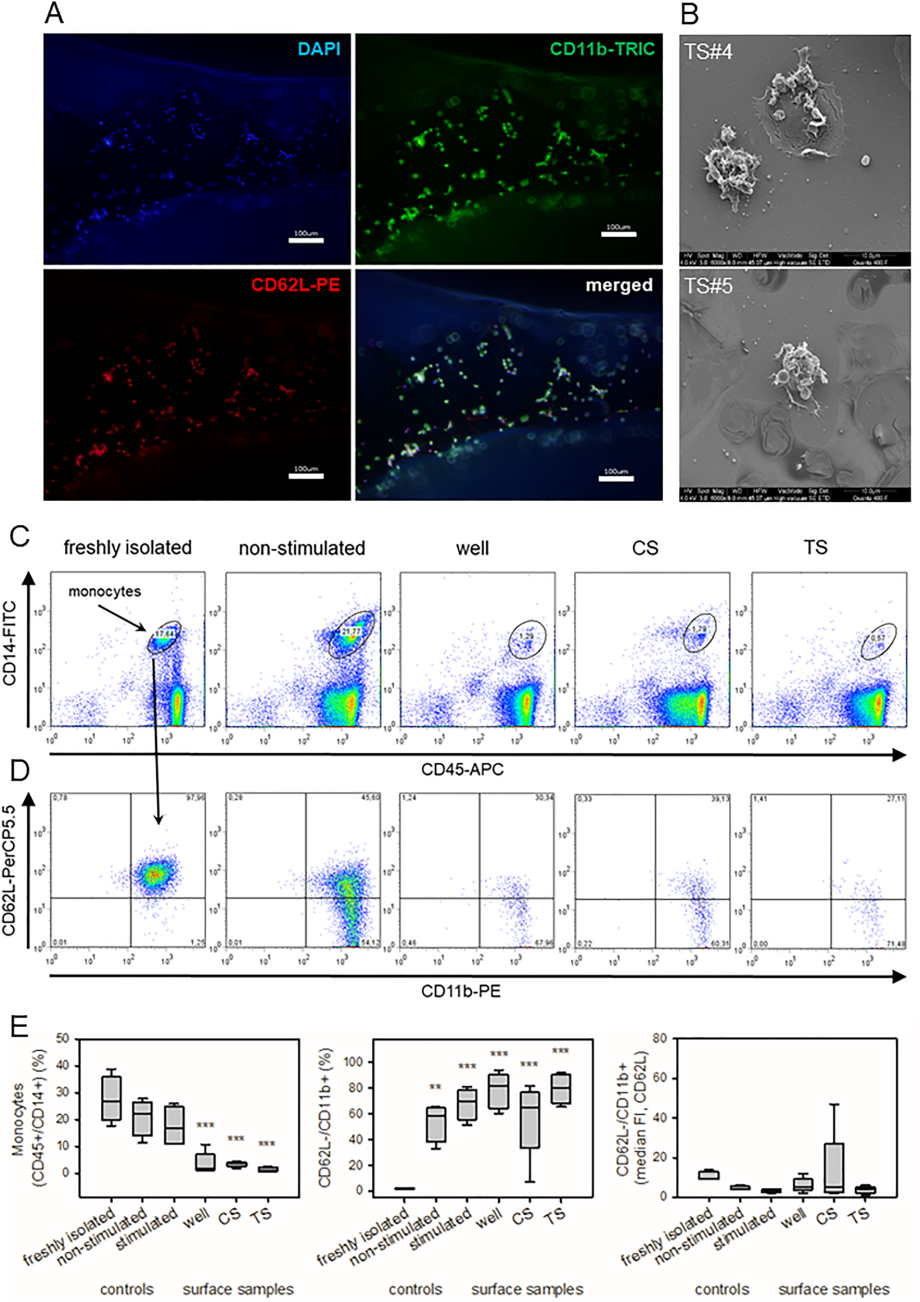
Response of PBMCs after contact with test samples (TS). Isolated PBMCs were cocultured with surface samples for 3 h and visualized using fluorescence microscopy (A) and SEM (**B**). **A** Representative image of PBMCs on TS with low cell density (blue, DAPI, nuclear staining), high expression of CD11b-FITC (green) and CD62L-PE (red). The border areas appeared blurred due to non-planar surface of TS (scale bar, 100 µm). **B** Representative SEM images of two TS batches with microaggregates consisting of monocytes, activated platelets and neutrophils. PBMCs in the supernatant were analyzed using FACS (**C**–**E**). **C** Identification of monocytes (CD45 + /CD14 + ) and lymphocytes (CD45 + /CD14-). **D** Monocytes were analyzed for their expression of CD11b and CD62L. Freshly isolated PBMCs included high percentage of monocytes that decreased in the supernatant of the surface samples (well, CS, TS). Fluorescence intensity (FI) of freshly isolated monocytes was high for CD11b (activated) and CD62L (non-activated). After 3 h of storage (non-stimulated), monocytes lost their CD62L signal (independent of the additional contact with surface samples). **E** Quantification of the proportion of monocytes (left), the proportion of CD62L-/CD11b+ cell population (middle) and its median FI (right). Data are median (IQR) of 5 independent experiments. Statistics (one-way ANOVA) compared to freshly isolated cells (**p < 0.01; ***p < 0.001)

PBMCs were incubated with surface samples (CS, TS) to determine the surface coverage using IF techniques and SEM. PBMCs adhered on TS and were positive for CD11b and CD62L. The additional stimulation with TNF/fMLP did not result in an increased CD11b and a decreased CD62L signal (data not shown). Therefore, no material-induced stimulation of adherent PBMCs was quantifiable (Fig. 6A). SEM analysis presented small microaggregates consisting of monocytes, platelets and neutrophils (Fig. 6B). Quantification of surface coverage on SEM samples was impossible due to low numbers of adherent PBMCs.

Non-adherent PBMCs in the supernatant presented a significant decrease in the proportion of monocytes (CD45 + /CD14 + ) (Fig. 6C, E) independent of the type of surface sample. Freshly isolated monocytes presented a high CD11b signal (stimulated) and a high CD62L signal (not stimulated) (Fig. 6D). Within the next 3 h (non-stimulated), more than 50% of the gated monocytes lost their CD62L signal (CD62L-/CD11b + ), which represents an additional stimulation of the monocytes. The three-hour contact of monocytes with different surface samples also resulted in an increase in the proportion of CD62L-/CD11b+ (and a decrease in the median fluorescence intensity of the CD62L signal) according to non-stimulated monocytes (Fig. 6E). Therefore, no statement could be made regarding material-induced stimulation of non-adherent monocytes.

#### Immunological response of PMNs with additive manufactured metal stents – NET-formation

**Fig. 7:**
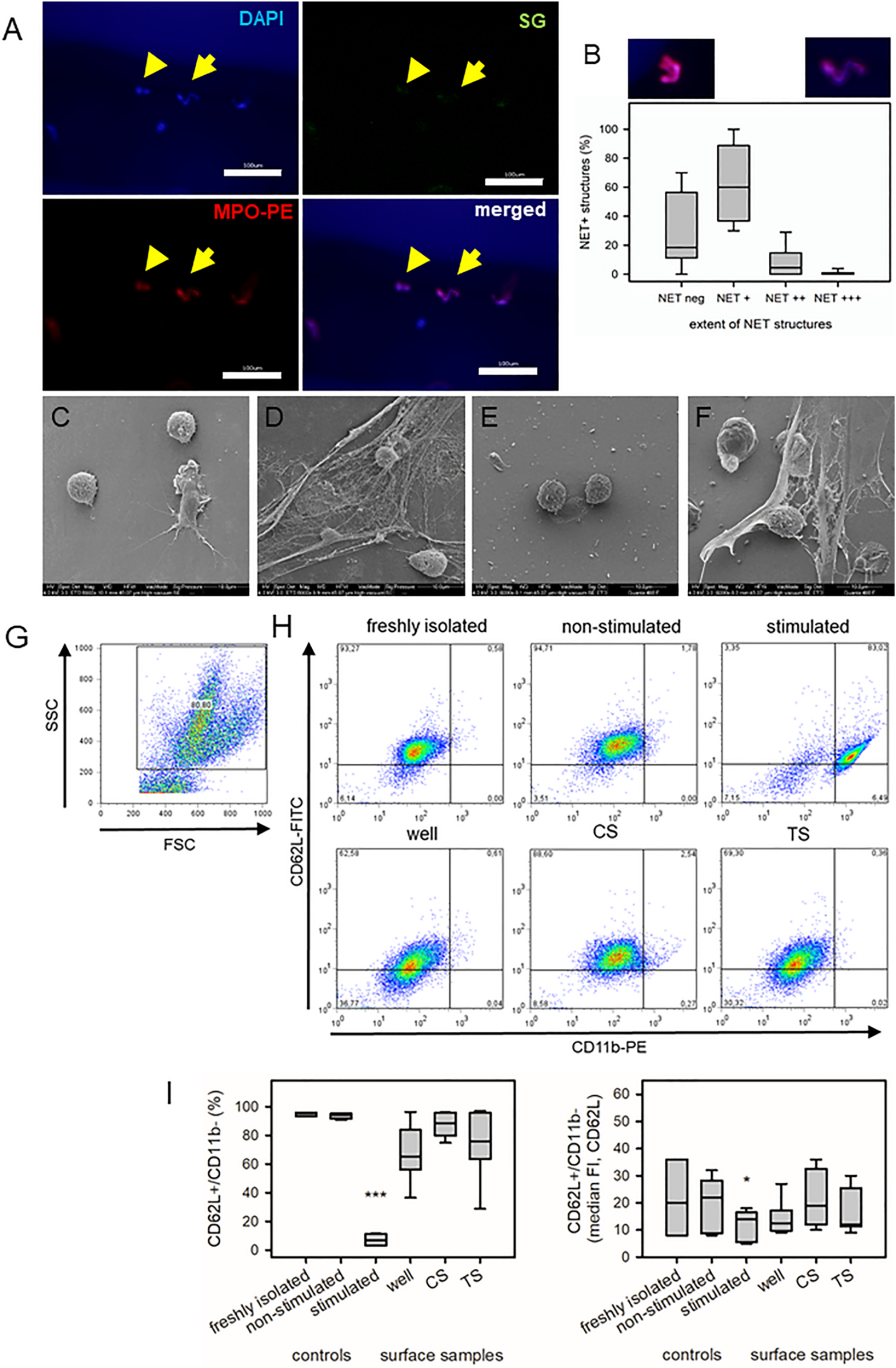
Response of PMNs after contact with surface samples – NET-formation. Isolated PMNs were cocultured with surface samples for 3 h and visualized using fluorescence microscopy (**A**, **B**) and SEM (**C**–**F**). **A** Representative image of PMNs on TS with low cell density (blue, DAPI, nuclear staining), marginal expression of SytoxGreen (SG, green) and MPO-PE (red, myeloperoxidase). The border areas appeared blurred due to non-planar surface of TS. Only clearly definable structures were included in the evaluation (scale bar, 100 µm). Arrow head identified a non-affected nucleus, arrow a typical NET (neutrophil extracellular trap) structure. **B** Differentiation of the shape of NET structures (NET neg, non-affected normal nuclei; NET + to +++, minimal alterations up to complete destruction of nuclei) and quantification of these stages relative to the total cell count. Representative SEM images of adherent PMNs (**C**–**F**) on glass cover slips (CS) with normal nuclear shape (**C**) and NET-formation after PMA incubation (**D**). **E** Sample of adherent PMNs on TS (**E**) with partial detection of NETs (**F**). Due to low cell density, SEM images were not quantified. PMNs in the supernatant were analyzed using FACS (**G**–**I**). **G** Identification of neutrophils according to forward (FSC) and side scatter (SSC) signal (gating) and (**H**) detection of the surface expression of CD11b and CD62L. Upper line, freshly isolated PMNs expressed moderate CD11b and high CD62L, a signal that remained unchanged after 3 h (non-stimulated). Stimulation with TNF/fMLP resulted in an increased proportion of cells with high CD11b and CD62L, and a lower proportion of cells that lost CD62L with remaining CD11b signal. Lower line, PMNs from the supernatant of surface samples: Contact with the well and TS resulted in a shift of the CD62L (decreased FI) signal. **I** Quantification of the proportion of CD62L + /CD11b- population (left) and its median FI (right). Data are median (IQR) of 5 independent experiments. Statistics (one-way ANOVA) compared to freshly isolated cells (**p < 0.01; ***p < 0.001)

PMNs were incubated with surface samples to determine the surface coverage and the extent of NET-forming using IF techniques and SEM (Fig. 7A-F). Small amounts of PMNs adhered on the surface of different surface samples (median (IQR), DAPI+ nuclei/mm^2^, 25 (12–48); MPO+ PMNs/mm^2^, 14 (7–32)). NET-structures consisted of short to long DNA-strands (NET + /++) up to web-like DNA structures (NET + ++). Sixty-four percent of the images presented short DNA-strands (NET + ). Extended NET structures were only detected in 0.8% of the IF-images (Fig. 7B). SEM presented also low surface coverage with PMNs (Fig. 7E). NET-structures could be detected only in isolated places (Fig. 7F).

Freshly isolated PMNs were CD62L + /CD11b- ( = non-stimulated) that remained unchanged up to the end of the experiment (3 h, non-stimulated) (Fig. 7H). The expression of CD11b was unchanged for all samples (except stimulation with TNF/fMLP). In contrast, contact of the PMNs with the polystyrene well led to a decrease in the expression of CD62L, which suggests PMN activation. However, this was attributed to the contact of PMNs with the well. Contact with CS and TS did not lead to any further stimulation of the PMNs in the supernatant (Fig. 7H, I).

## Platelet adhesion and activation after contact with additive manufactured metal stents

Platelet adhesion tests were utilized to determine the hemocompatibility of additive manufactured metallic stent materials. After incubation with isolated platelets (PRP), platelet adhesion/activation was verified using IF-methods (staining with RP, staining with CD61 and CD62P-antibodies) and SEM.

**Fig. 8:**
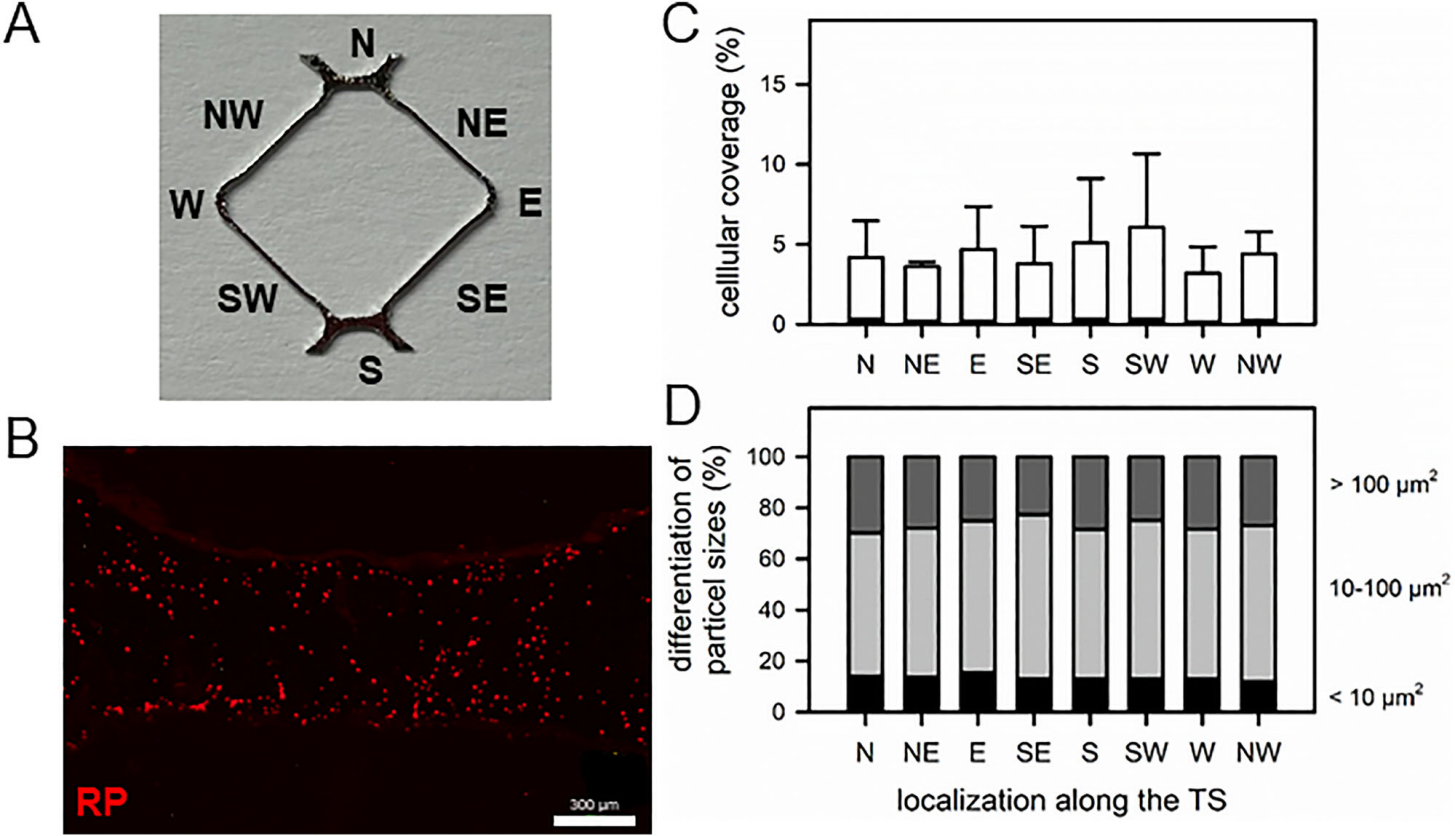
Platelet adhesion on TS surfaces using rhodamine-phalloidine (RP) staining. Isolated platelets were cocultured with TS for 1 h, stained with RP and visualized using immunofluorescence microscopy. **A** Orientation along the TS (divided into cardinal directions). **B** Representative image of adherent RP-stained platelets on TS. Due to non-planar surface geometry, the edge regions of the samples appear blurred (scale bar 300 µm). **C** Quantification of the cellular coverage (area of RP-stained platelets relative to the analyzed surface area). Data are median (IQR) of 5 independent experiments. **D** Differentiation of particle sizes (in % of total cellular coverage)

Staining cells cytoskeleton with rhodamine-phalloidin revealed the presence of focal adhesions and organized intracellular actin network, supporting the existence of an adhesion process [37]. Platelets adhered evenly along the surface of the TS (Fig. 8B). The cellular coverage ranged between 0.5 and 10% (Fig. 8C). The colonization and distribution of the particle sizes were independent of the localization on the rhombic TS (struts, corners) (Fig. 8D). More than 50% of adherent platelets presented particle sizes between 10 and 100 µm^2^.

**Fig. 9:**
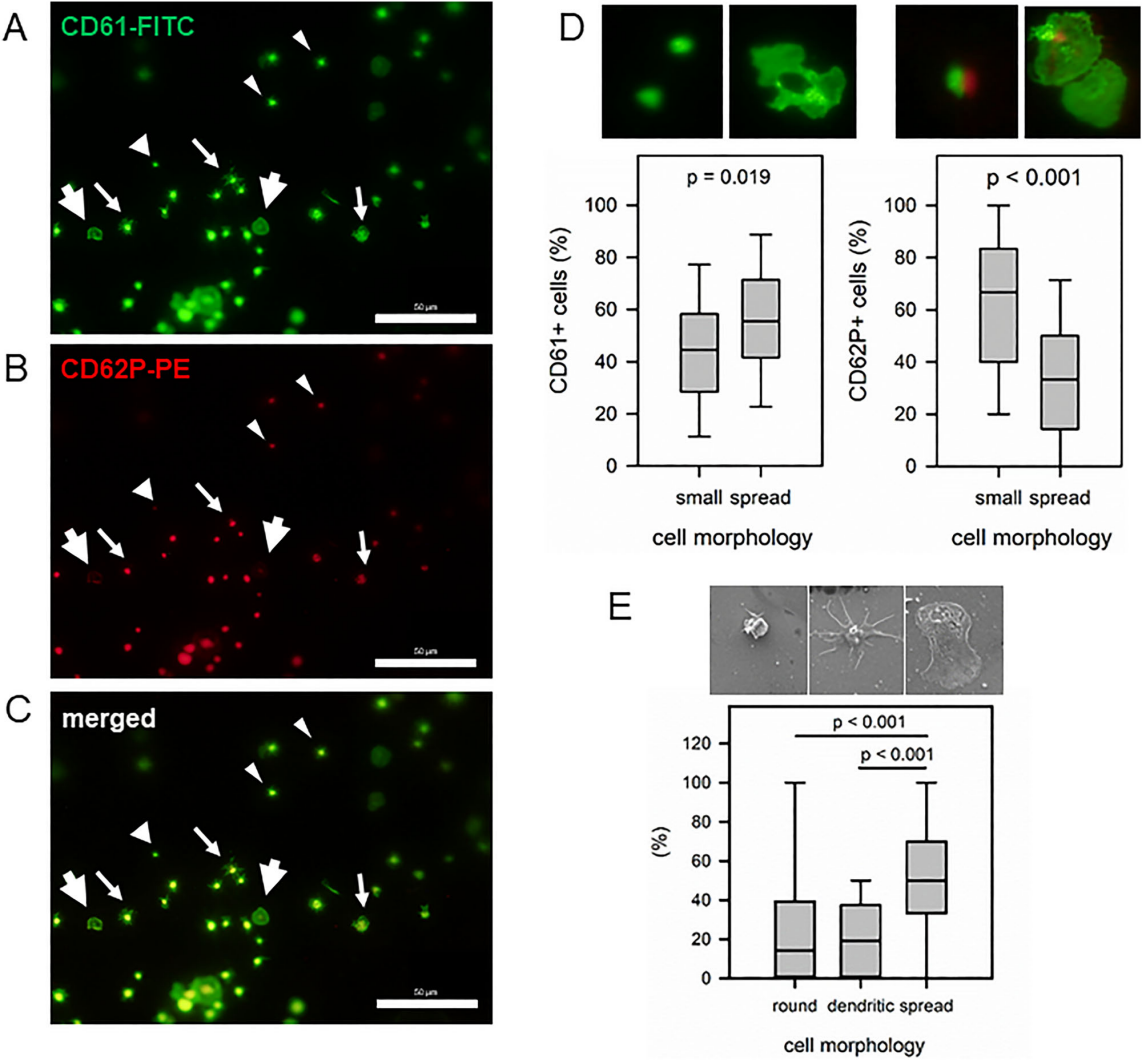
Morphology of adherent platelets on TS surfaces using CD61-FITC and CD62P-PE antibody staining (**A**–**D**) and SEM (**F**). Isolated platelets were cocultured with TS for 1 h. **A**–**C** Representative antibody stainings. Bright arrow head, small cells without CD62P-signal; small arrow head, small cells with CD62P-signal; bright arrow, spread cells without CD62P-signal; small arrow, spread cells with CD62P-signal (scale bar, 50 µm). Due to non-planar surface geometry, the edge regions of the samples appear blurred and were excluded from evaluation. **D** Quantification of respective cell populations with representative cell morphology in the header. **E** Quantification of different cell morphologies of adherent platelets on TS using SEM. Data are median (IQR). Statistics (one-way ANOVA) with p-values as indicated

IF staining with a CD61-FITC-antibody enabled visualization of different cell morphologies (small and spread cells). However, due to the non-planar surface of the TS, only parts of the structures could be clearly demarcated. Overall, the proportion of spread cells was significantly higher compared to small cells (*p* = 0.019) (Fig. 9A–D). Furthermore, more than 60% of the small cells were CD62P-positive, while only about 40% of the spread cells were CD62P-positive.

Finally, SEM analysis were used to differentiate the morphology of adhered platelets on TS. Only 3 of the 5 production patches allowed the quantification of cell density (cell count per 0.02 mm^2^, 3000x magnification) and different platelet morphologies (round, dendritic, spread) [38]. Platelet density was 6.4 ± 9.2, 18.6 ± 6.5 and 25.1 ± 16.5 platelets/0.02 mm^2^ identified on patch #5, #4, and #2, respectively. Only 3 morphologies were differentiated with significantly higher amounts of spread cells (Fig. 9E). This was in agreement with IF data (Fig. 9D).

**Fig. 10:**
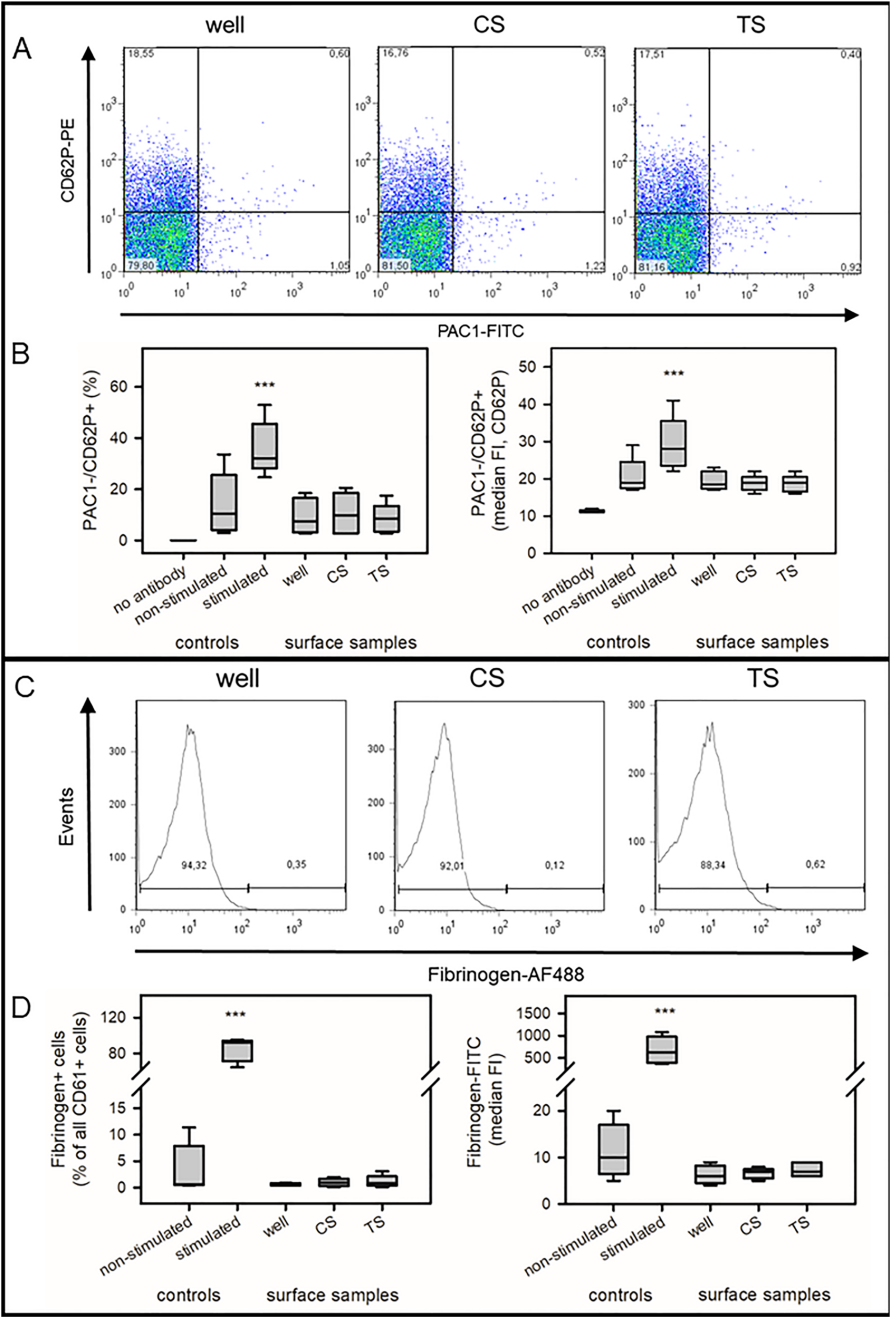
Activation status of platelets in the supernatant after contact with surface samples. Platelets in the supernatant were analyzed using FACS analysis. **A**, **B** Expression of PAC1 and CD62P was comparable after contact with all surface samples (well, CS, TS). **B** Quantification of alterations in the proportion of PAC1-/CD62P+ cells and its median fluorescence intensity (FI). Only stimulation of platelets with ADP resulted in a significant increase in both parameters (***p < 0.001). **C**, **D** Identification of fibrinogen-AF488 binding to CD61-PE-positive platelets. **C** The contact with surface samples did not induce an increase in the fibrinogen-AF488 signal. **D** Quantification of the proportion of platelets that bind fibrinogen-AF488 relative to all CD61-positive platelets and its median fluorescence intensity. Only stimulation with ADP resulted in a significant increase in both parameters (***p < 0.001)

Non-adherent platelets in the supernatant were classified according to their activation status (staining with antibodies: PAC1, CD62P, CD61; fibrinogen-FITC binding). Ten to thirty percent of the non-stimulated cells (no surface contact) were CD62P-positive (presumably due to platelet isolation) (Fig. 10A, B). PAC1 was not detectable. Stimulation with ADP resulted in significant upregulation of CD62P and PAC-1 (Supplementary Fig. S1). Contact of platelets with the surface samples did not affect their expression of CD62P and PAC1 (Fig. 10A, B). The PAC1-/CD62P+ platelets presented a significant upregulation of CD62P after ADP stimulation, while CD62P expression was unchanged after surface contact. In addition, CD61-positive platelets were examined for fibrinogen binding activity (Fig. 10C, D). Treatment of platelets with ADP increased fibrinogen binding (Supplementary Fig. S1), while contact with the surface samples did not affect fibrinogen binding (Fig. 10C, D).

## Discussion

The mechanical properties of additively manufactured and surface-modified 316 L stainless steel stents are comparable to conventional metallic stents [22]. Data on hemocompatibility of additive manufacturing and post-processing failed so far. The present study demonstrated that the additively manufactured metallic stents were not cytotoxic, non-hemolytic, presented low adhesion of blood cells, and showed no material-induced stimulation of circulating leukocytes and platelets.

This study approved the existence of the typical process-related geometric irregularities of additively manufactured 316 L stainless steel stents [22]. Their high surface roughness, deviation in the strut cross-sectional shape and the strut diameter, strut waviness, tapers, and porosity [22, 39–41] are attributed to the layer-by-layer manufacturing, local energy input and melting of the powder, different heat dissipation properties of the powder and the solidified material, as well as L-PBF process parameters [42–44]. Post-processing steps as described in the present study have an impact on the mechanical response of L-PBF stents [22].

### Heat treatment homogenized the microstructure of additive manufactured stents

Heat treatment at a temperature of 1100 °C for 1 h and subsequently cooling inside the furnace (1) reduced yield strength to improve plastically deformability (sequential expansion of the aortic stenosis using a balloon catheter), (2) increased tensile strength to avoid material failure (the aortic stent), and (3) homogenized microstructure to optimize mechanical properties. This procedure should also be accompanied by sufficient elongation at break to ensure deformability during expansion [22]. The microstructure of the additively manufactured 316 L in the AB state shows overlapping melt pools, coarse elongated grains and δ -ferrites ( = microscopic honeycomb-like substructure) [34, 45]. δ-ferrite is a strength-enhancing second phase which is produced by high rates of heating (melting) and cooling (solidification) during the manufacturing via L-PBF and with accompanying high density of dislocations and residual stress in the material [46, 47]. During the heat treatment process the density of dislocations and the residual stress are reduced and the dislocations act as nucleation points for recrystallisation [48]. The recrystallized microstructure is characterized by more equiaxed coarse grains (Fig. 3) resulting in reduced yield strength (Table 3) [46, 48, 49]. In addition to recrystallization, the heat treatment of L-PBF 316 L is associated with the dissolution of the δ-ferrite phase as a reinforcing second phase [45], which explains the reduction in the yield strength and which is a prerequisite for the material to be used as an implant [46], DIN EN ISO 5832-1). Therefore, it could be assumed that heat treated additively manufactured stents are sequentially easier to deform using a balloon. In addition, this post-treatment makes the material (the struts and the aortic stent) more resistant to failure due to greater forces than the AB state. The consistently high deformability and disappearance of the δ -ferrite conclude the first point of post-processing and pave the way for the use of the material as a stent.

### Electropolishing smoothed the surface of the additive manufactured stents

Electropolishing reduced high surface roughness Sa (an extension of the line roughness Ra) of additively manufactured structures resulting in a target value of less than 1.0 µm [23, 50]. As shown in Table 4, for the build and post-processed stent struts a comparable roughness value Ra is reached. The relatively high standard deviation of the roughness seen is due to pores that can be exposed by an electropolishing process [51]. The literature provides valuable insight into the effect of electropolishing on various aspects of 316 L stainless steel, including surface roughness, passivation properties, wettability and biocompatibility. Studies have shown that electropolishing besides the significant reduction of surface roughness, resulting in a smoother surface [51, 52], also altered surface energies which have shown an influence on the adhesion of bacteria [53]. In contrast, the effect of alterations in the surface energies on the adhesion of endothelial cells or fibroblasts is controversial discussed. Latifi et al (2013) showed an increase of the surface energy from conventional medical-grade 316 L stainless steel after electropolishing from 30 mJ/m^2^ to about 50 mJ/m^2^ without alteration of cell growth characteristics. In the present study, additive-manufactured 316 L stainless steel samples without (AB) and with post-treatments (TS) showed a higher surface energy (55 mJ/m^2^ and 61 mJ/m^2^) compared to conventional 316 L samples of Latife et al. (2013). Since the values are above the critical surface energy for biomaterials ( > 55 mJ/m^2^) [25], the surface of additive manufactured 316 L samples have cell-adhesive properties as shown in the present study.

In addition, electropolishing promotes the formation of a passive oxide layer, particularly a chromium-rich oxide layer, which improves corrosion resistance and long-term durability of implants. [54] have shown that a chromium-rich passive oxide layer, which is thicker than the natural one, is formed by electropolishing for different cell currents. The process also increased biocompatibility and hemocompatibility [54]. High surface roughness of nanoporous stainless steel particles induced high platelet adhesion and smooth muscle cell adhesion [55]. However, there are opposing data on the correlation of surface roughness of stainless steel and bacterial adhesion [56–58]. Rougher samples analyzed from [59] also showed an optical density of over 80%, which roughly corresponds to the roughness investigated here. Regarding cell adhesion, the aim of future investigations could be to reduce the surface roughness even further.

### Surface modified of additive manufactured stents presented adequate biological responses

Before a surface can be implemented in a biomedical device, it must be shown to be non-cytotoxic. The biomedical-grade 316 L SS used in the present study has been used for biomedical applications for a long time and is characterized by good hemocompatibility and corrosion resistance. To reveal the influence of additive manufacturing on stents and their specific post-processing steps on the biological response, we performed in vitro studies with isolated blood cells.

Neither the additive manufacturing process nor the various surface treatments released any toxic metal ions that affected cell morphology or the metabolic activity within 3 days of cultivation. According to ISO 10993-5, cell viability of L929 cells for experimental materials were more than 70%, indicating that the additive manufactured (AB) as well as all post-processed samples were non-cytotoxic to L929. [60] conducted similar experiments and claimed that scanning speed had strong effects on the in vitro cytotoxicity of 316 SS parts elaborated by selective laser melting (L-PBF). However, the difference between highest and lowest effect (900 vs. 800 mm/s scanning speed) was only 22% and this means that the value is still within the tolerance range of 30% (according to ISO 10993-5). In addition, direct contact cytotoxicity (ISO 10993-5) was performed. The surface of post-processed additive manufactured stents (TS) was completely covered with cells which indicates good biocompatibility.

Furthermore, the hemolysis rates were well within the 5% hemolysis ratio that is considered acceptable for blood material compatibility (ASTM F756-08 F756-00, A., Standard Practice for Assessment of Hemolytic Properties of Materials, 2000, ASTM International West Conshohocken, PA, USA, 2000; Li et al. 2001). Good hemocompatibility in form of low hemolysis rates ( < 1%) of 316 L stainless steel as well as surface modifications (e.g. plasma polymer-like allylamine films) were approved by [61]. Furthermore, process parameters such as scanning speed during L-PBF of 316 L did not affect hemolysis induction [60].

Leukocyte adhesion to artificial surfaces is an important phenomenon in the evaluation of biomaterials because adherent leukocytes are often related to the inflammatory response seen after implantation (review from [62]). Contact of the additive-manufactured stent with isolated leukocytes resulted in a moderate adhesion of PMNs and monocytes on the metallic surface as well as partial NET formation as shown by SEM and IF. In vitro evaluation of leukocyte adhesion and NET formation to stent material was connected with its proinflammatory properties [63]. However, the underlying mechanism remains unclear. Initially plasma proteins (in particular complement factor iC3b and fibrinogen) adsorbed accompanied by platelet and leukocyte binding [62]. In the present study, metallic surfaces were pre-coated with BSA and AB-serum [64]. Albumin was chosen since it is abundantly present in blood and at sites of injury and because it is known to „passivate“ biomaterial surfaces, blunting proinflammatory and prothrombogenic responses [65, 66]. AB-serum was used as a source of both albumin and fibrinogen. While there were no specific ligands for PMN-binding to albumin [67], the ß2-integrin Mac-1 and the intercellular adhesion molecule ICAM-1 mediate leukocyte binding to fibrinogen [68] to cause proinflammatory effects at implant surfaces, mainly by causing an increased recruitment and adhesion of leukocytes in inflammation and tissue repair at implant surfaces [68]. Protein adsorption was not tested in the present study. Furthermore, the contact of leukocytes with artificial surfaces (such as cardiovascular devices) resulted in L-selectin (CD62L) shedding and CD11b upregulation on circulating leukocytes. However, in the present study, the isolation process already led to a stimulation (CD11b) of the cells. These cells adhered onto the samples and presented high intensity of CD11b and indifferent CD62L-signals. This response cannot be attributed to the adhesion process onto the metallic samples alone.

Different platelet adhesion tests were introduced to determine the hemocompatibility of additive manufactured metallic stents. Staining cells cytoskeleton with rhodamine-phalloidin revealed the presence of focal adhesion and organized intracellular actin network, supporting the existence of an adhesion process [37]. This is a simple method to visualize platelets and allowed semi-automated quantification. After adherence and fixation, cells were only stained with the fluorescent dye without more processing procedures that ensured realistic cell adhesion. Only 0.5 to maximum 10% of randomly selected TS areas were covered with single cells or microaggregates. Platelets were uniformly distributed on TS. However, actin staining does not allow an assessment of the activation state of the cells. When platelets are activated they change morphologies by forming dendrites that attach to the surface and other platelets [59]. This activation is a prerequisite to blood-clotting and thrombosis [69]. Platelet activation was characterized through SEM imagery. SEM analysis of adherent platelets on additive-manufactured stents presented a cell density that ranged between 6 and 25 platelets per 0.02 mm^2^ and adherent platelets were mainly fully spread. Cell density corresponds with samples made of high nitrogen nickel-free austenitic stainless steel and nitinol alloy using the same adhesion protocol (1 h incubation with PRPs) [35]. However, [35] did not subdivide the shape of adherent platelets – all cells were altered in shape with pseudopodia. A direct comparison of the cell density with other studies that also work with 316 L was not possible because the cell density depends on the number of platelets used for adhesion testing. Most studies compared uncoated 316 L surfaces with different coatings (such as heparin, titanium films, different alloys) and presented abundant platelet adhesion with high cell density consisting of mainly dendritic or spread dendritic cells on 316 L metallic sheets [59, 61, 70]. In contrast, platelet deposition on perfused polylactide (PLA) stents was significantly higher compared to stainless steel stents [71]. In most studies, SEM was used to demonstrate shape alterations and thus to calculate the activation status of adherent platelets. However, this method is time-consuming, expensive and required a special equipment. An alternative to SEM is IF using specific fluorophore-labeled platelet antibodies (e.g., CD61). The present study demonstrated a clear differentiation of small and spread platelets using a FITC-anti-human CD61 antibody. The majority of adherent platelets on additive manufactured stents were fully spread compared to small and bright green cells. This corresponds to SEM data. However, the additional staining with a PE-anti-human CD62P antibody allowed the colocalization of CD61 and the activation marker. It was shown, that more than 70% of small cells expressed CD62P, while only 30% of the spread cells presented the CD62P signal. In particular, the high number of small cells that express CD62P suggests that not only a change in shape but also the expression of activation markers must be taken into account when estimating the activation status of adherent platelets. The CD62P staining appeared as a punctuate expression both on small and spread cells. The CD62P antigen is a member of the selectin family of cellular adhesion molecules located in the storage granules of platelets. Upon activation, CD62P is released and stably expressed on the surface of activated platelets [72]. Visualization of adherent/activated platelets with fluorophore-labeled antibodies were also shown in a study of [73]. Platelets adhered on silicon and polysilicon that were identified by the platelet marker CD41 and CD62P, while polymer-conjugated silicon substrates reduced platelet adhesion. ADP-stimulated platelets on silicon samples not only expressed CD41 and CD62P but also changed their morphology (spread). In summary, while platelet adhesion on surface modified additive manufactured stents was low ( < 10% surface coverage), the majority of adherent cells presented spread cell morphology and/or extended CD62P expression. However, these data did not allow a statement about an increased risk of thrombosis.

In contrast, circulating platelets in the supernatant of the adhesion experiments showed no increased expression of platelet activation markers or increased fibrinogen binding activity. The lack of PAC1-expression on the platelet surface suggested that the fibrinogen-receptor complex (GPIIb/IIIa) was not activated. Therefore, fibrinogen binding remained unchanged. There are various possible explanations for the absence of membrane-bound CD62P on circulating cells. We suggested that the adhesion by itself activates platelets and resulted in an increased expression of CD62P. However, the remaining and non-adherent platelets in the supernatant are either non-activated or they degranulate and release the soluble CD62P from alpha-granules to the supernatant. A suspected non-activated state of circulating platelets could be detected by subsequent testing for activation potential (e.g., by thrombin). There is also evidence that the circulating cells also express CD62P, but rapidly (within 10 min) lose surface P-selectin to the plasma pool [74]. However, in the present study, neither the activation potential of the circulating cells nor the concentration of soluble CD62P in the supernatant were determined. A correlation of the different expression forms of CD62P failed so far. In most clinical and experimental studies only one detection method is used to demonstrate platelet activation [75]. The accuracy of this procedure was shown in selected experimental studies. The contact of blood with test materials (60 min, 37 °C, static conditions) and the detection of soluble CD62P allowed the differentiation of the thrombogenic potentials of the materials (high > intermediate > low, glass beads > 316 stainless steel sheets > polytetrafluoroethylene cord) [76]. Gemmell et al. [77] used the same experimental setup to demonstrate that the contact of blood with various polymeric surfaces resulted in an increase in microparticles and CD62P-positive circulating platelets accompanied by a doubling of soluble CD62P levels. Increased release of soluble CD62P was observed after contact of blood with different metallic materials (e.g. L605, 316 L SS) compared to blood without material contact [78]. However, the used experimental conditions did not allow a differentiation of the tested metal surfaces.

While static test systems are simple and cost-effective methods and allow first insights in the hemocompatibility of biomaterials, there are major limitations such as cell sedimentation and the big blood-air interface, which can lead to a protein aggregation and result in false positive platelet activation. Furthermore, an important influencing factor for medical devices – shear forces – is neglected [79]. The introduction of plasma-activated coatings onto 316 L SS stents was investigated in a modified Chandler loop. Under an approximate coronary flow of 85 mL/min, a significant increase in the P-selectin concentration was only detected after contact of uncoated stents with blood [80]. Dynamic studies of additively manufactured 316 L SS stents using L-PBF are still lacking and were not investigated in the present study. Therefore, a comparison with relevant literature is provided in Table 5 to contextualize the present findings.

**Table 5:** Main features of additively manufactured 316 L stents compared to reported literature

Characteristic	Results from the present study	Reported literature
Manufacturing & post-processing	L-PBF, heat treatment, chemical etching, electropolishing	Conventional and AM 316 L with various post-processing routes [22, 39–41]
Microstructure after heat treatment	Uniform grain structure, no visible melt pools	Grain homogenization after heat treatment [45–49]
Mechanical response	reduced yield strength, increased tensile strength, unchanged ductility	Comparable trends reported for heat-treated AM 316 L [22, 46]
Surface roughness (Ra)	Reduced to < 1 µm	Similar reduction after electropolishing [23, 50–52]
Surface energy	Increased; exceeding the critical value for cell adhesion	Increase after electropolishing [25, 53]
Cytotoxicity (ISO10993-5)	Non-cytotoxic ( > 70% viability)	Non-cytotoxic behavior of 316 L [60]
Hemolysis	< 1%	Low hemolysis of 316 L surfaces [61]
Leukocyte / PMN response	Moderate adhesion, no clear material-induced stimulation	Comparable responses under static conditions [62, 63]
Platelet adhesion	Low surface coverage (0.5–10%), 6–25 platelets per 0.02 mm^2^	Higher platelet adhesion on unmodified 316 L compared to coated metal surfaces [59, 61, 70]
Platelet activation	Shape alteration (spread) of 80% of adherent platelets, CD62P expression of small as well as spread adherent platelets, no activation of circulating platelets	Higher expression of CD62P of adherent platelets on unmodified 316 L compared to coated metal surfaces [59, 61, 70]

## Conclusions

The main advantage of the presented approach lies in combining the design freedom and rapid customization enabled by additive manufacturing with tailored mechanical properties and satisfactory biological performance achieved through targeted post‑processing. This enables the production of individualized metallic stents within short fabrication times while maintaining material and biological requirements. However, the study is limited by the use of static in‑vitro test conditions that do not fully reflect the dynamic flow and shear stresses of the cardiovascular system, and long‑term performance as well as in‑vivo responses were not investigated. Overall, the findings support the potential of additively manufactured and post‑processed 316 L stainless steel stents as a promising platform for personalized cardiovascular implants, while further studies under dynamic and in‑vivo conditions are required to confirm long‑term safety and clinical applicability.

## Supplementary information

Supplementary information

Supplementary information

## References

[1] Verscheure D, Haulon S, Tsilimparis N, Resch T, Wanhainen A, Mani K, et al. Endovascular Treatment of Post Type A Chronic Aortic Arch Dissection With a Branched Endograft: Early Results From a Retrospective International Multicenter Study. Ann Surg. 2021;273:997–1003. 10.1097/SLA.0000000000003310. 30973389 10.1097/SLA.0000000000003310

[2] Law, Kölbel Y, Detter T, Rohlffs C, Kodolitsch F, von Y, et al. Emergency Use of Branched Thoracic Endovascular Repair in the Treatment of Aortic Arch Pathologies. Ann Thorac Surg. 2019;107:1799–806. 10.1016/j.athoracsur.2018.09.020. 30389447 10.1016/j.athoracsur.2018.09.020

[3] Porterie J, Hostalrich A, Dagenais F, Marcheix B, Chaufour X, Ricco J-B. Hybrid Treatment of Complex Diseases of the Aortic Arch and Descending Thoracic Aorta by Frozen Elephant Trunk Technique. J Clin Med. 2023. 10.3390/jcm12175693. 37685761 10.3390/jcm12175693 PMC10488597

[4] Suto Y, Yasuda K, Shiiya N, Murashita T, Kawasaki M, Imamura M, et al. Stented elephant trunk procedure for an extensive aneurysm involving distal aortic arch and descending aorta. J Thorac Cardiovasc Surg. 1996;112:1389–90. 10.1016/S0022-5223(96)70157-5. 8911340 10.1016/S0022-5223(96)70157-5

[5] Leone A, Beckmann E, Martens A, Di Marco L, Pantaleo A, Reggiani LB, et al. Total aortic arch replacement with frozen elephant trunk technique: Results from two European institutes. J Thorac Cardiovasc Surg. 2020;159:1201–11. 10.1016/j.jtcvs.2019.03.121. 31208809 10.1016/j.jtcvs.2019.03.121

[6] Yoshitake A, Tochii M, Tokunaga C, Hayashi J, Takazawa A, Yamashita K, et al. Early and long-term results of total arch replacement with the frozen elephant trunk technique for acute type A aortic dissection. Eur J Cardiothorac Surg. 2020;58:707–13. 10.1093/ejcts/ezaa099. 32236552 10.1093/ejcts/ezaa099

[7] Ogino H, Okita Y, Uchida N, Kato M, Miyamoto S, Matsuda H, et al. Comparative study of Japanese frozen elephant trunk device for open aortic arch repairs. J Thorac Cardiovasc Surg. 2022;164:1681–.e2. 10.1016/j.jtcvs.2021.03.079. 33965229 10.1016/j.jtcvs.2021.03.079

[8] Tian DH, Ha H, Joshi Y, Yan TD. Long-term outcomes of the frozen elephant trunk procedure: a systematic review. Ann Cardiothorac Surg. 2020;9:144–51. 10.21037/acs.2020.03.08. 32551246 10.21037/acs.2020.03.08 PMC7298235

[9] Revilla-León M, Meyer MJ, Özcan M. Metal additive manufacturing technologies: literature review of current status and prosthodontic applications. Int J Comput Dent. 2019;22:55–67. 30848255

[10] Kunčická L, Kocich R, Lowe TC. Advances in metals and alloys for joint replacement. Prog Mater Sci. 2017;88:232–80. 10.1016/j.pmatsci.2017.04.002.

[11] Ferraiuoli P, Taylor J, Martin E, Fenner J, Narracott A. The Accuracy of 3D Optical Reconstruction and Additive Manufacturing Processes in Reproducing Detailed Subject-Specific Anatomy. J Imaging. 2017;3:45 10.3390/jimaging3040045.

[12] Băilă D-I, Păcurar R, Savu T, Zaharia C, Trușcă R, Nemeș O, et al. Mechanical and Wetting Properties of Ta2O5 and ZnO Coatings on Alloy Substrate of Cardiovascular Stents Manufactured by Casting and DMLS. Mater (Basel). 2022. 10.3390/ma15165580. 10.3390/ma15165580 PMC9412485 36013717

[13] He R, Langi E, Garrard R, Attallah MM, Silberschmidt VV, Vogt F, et al. In silico evaluation of additively manufactured 316L stainless steel stent in a patient-specific coronary artery. Med Eng Phys. 2022;109:103909 10.1016/j.medengphy.2022.103909. 36371086 10.1016/j.medengphy.2022.103909

[14] Disegi JA, Eschbach L. Stainless steel in bone surgery. Injury. 2000;31:2–6. 10.1016/S0020-1383(00)80015-7. 11270076 10.1016/s0020-1383(00)80015-7

[15] Hosseinalipour SM, Ershad-langroudi A, Hayati AN, Nabizade-Haghighi AM. Characterization of sol–gel coated 316L stainless steel for biomedical applications. Prog Org Coat. 2010;67:371–4. 10.1016/j.porgcoat.2010.01.002.

[16] Muley SV, Vidvans AN, Chaudhari GP, Udainiya S. An assessment of ultra fine grained 316L stainless steel for implant applications. Acta Biomater. 2016;30:408–19. 10.1016/j.actbio.2015.10.043. 26518104 10.1016/j.actbio.2015.10.043

[17] Cox SC, Jamshidi P, Eisenstein NM, Webber MA, Burton H, Moakes RJA, et al. Surface Finish has a Critical Influence on Biofilm Formation and Mammalian Cell Attachment to Additively Manufactured Prosthetics. ACS Biomater Sci Eng. 2017;3:1616–26. 10.1021/ACSBIOMATERIALS.7B00336. 33429647 10.1021/acsbiomaterials.7b00336

[18] Baakili SE, Munyensanga P, Bricha M, Mabrouk KE. Porous Metallic Implants from Additive Manufacturing to Biocorrosion: A Review. Johns Matthey Technol Rev. 2024;68:71–90. 10.1595/205651324x16826780236175.

[19] Ghosh S, Indrakumar S, Ghosh S, Gopal V, Nilawar S, Manivasagam G, et al. Surface nanocrystallization enhances the biomedical performance of additively manufactured stainless steel. J Mater Chem B. 2023;11:9697–711. 10.1039/d3tb01534c. 37789772 10.1039/d3tb01534c

[20] Rajput AS, Kapil S, Das M. Surface Enhancement of Additively Manufactured Bone Plate Through Hybrid-Electrochemical Magnetorheological Finishing Process. 3D Print Addit Manuf. 2024;11:e1380–e1393. 10.1089/3dp.2023.0028. 39359582 10.1089/3dp.2023.0028 PMC11442415

[21] Al-Mamun NS, Mairaj Deen K, Haider W, Asselin E, Shabib I. Corrosion behavior and biocompatibility of additively manufactured 316L stainless steel in a physiological environment: the effect of citrate ions. Addit Manuf. 2020;34:101237. 10.1016/j.addma.2020.101237.

[22] Wiesent L, Schultheiß U, Lulla P, Noster U, Schratzenstaller T, Schmid C, et al. Computational analysis of the effects of geometric irregularities and post-processing steps on the mechanical behavior of additively manufactured 316L stainless steel stents. PLoS One. 2020;15:e0244463 10.1371/journal.pone.0244463. 33373392 10.1371/journal.pone.0244463 PMC7771678

[23] Grad M, Nadammal N, Schultheiss U, Lulla P, Noster U. An Integrative Experimental Approach to Design Optimization and Removal Strategies of Supporting Structures Used during L-PBF of SS316L Aortic Stents. Appl Sci. 2021;11:9176 10.3390/app11199176.

[24] Owens DK, Wendt RC. Estimation of the surface free energy of polymers. J Appl Polym Sci. 1969;13:1741–7. 10.1002/app.1969.070130815.

[25] Helsen JA, editor. Metals as biomaterials. Chichester, Weinheim: Wiley; 1998.

[26] Li W, Zhou J, Xu Y. Study of the in vitro cytotoxicity testing of medical devices. Biomed Rep. 2015;3:617–20. 10.3892/br.2015.481. 26405534 10.3892/br.2015.481 PMC4535150

[27] Motlagh D, Allen J, Hoshi R, Yang J, Lui K, Ameer G. Hemocompatibility evaluation of poly(diol citrate) in vitro for vascular tissue engineering. J Biomed Mater Res A. 2007;82:907–16. 10.1002/jbm.a.31211. 17335023 10.1002/jbm.a.31211

[28] Macuvele DLP, Colla G, Cesca K, Ribeiro LFB, Da Costa CE, Nones J, et al. UHMWPE/HA biocomposite compatibilized by organophilic montmorillonite: An evaluation of the mechanical-tribological properties and its hemocompatibility and performance in simulated blood fluid. Mater Sci Eng C Mater Biol Appl. 2019;100:411–23. 10.1016/j.msec.2019.02.102. 30948077 10.1016/j.msec.2019.02.102

[29] Sixt S, Gruber M, Kolle G, Galla T, Bitzinger D. The Effect of Local Anesthetics on Neutrophils in the Context of Different Isolation Techniques. Biomedicines 2023. 10.3390/biomedicines11082170. 10.3390/biomedicines11082170 PMC10452207 37626667

[30] Blix IJ, Helgeland K, Kähler H, Lyberg T. LPS from Actinobacillus actinomycetemcomitans and the expression of beta2 integrins and L-selectin in an ex vivo human whole blood system. Eur J Oral Sci. 1999;107:14–20. 10.1046/j.0909-8836.1999.eos107104.x. 10102746 10.1046/j.0909-8836.1999.eos107104.x

[31] Frank RD, Dresbach H, Thelen H, Sieberth H-G. Glutardialdehyde induced fluorescence technique (GIFT): a new method for the imaging of platelet adhesion on biomaterials. J Biomed Mater Res. 2000;52:374–81. 10951378 10.1002/1097-4636(200011)52:2<374::aid-jbm18>3.0.co;2-z

[32] Ko T-M, Lin J-C, Cooper SL. Surface characterization and platelet adhesion studies of plasma-sulphonated polyethylene. Biomaterials. 1993;14:657–64. 10.1016/0142-9612(93)90064-9. 8399962 10.1016/0142-9612(93)90064-9

[33] Lehle K, Li J, Zimmermann H, Hartmann B, Wehner D, Schmid T, et al. In vitro Endothelialization and Platelet Adhesion on Titaniferous Upgraded Polyether and Polycarbonate Polyurethanes. Mater (Basel). 2014;7:623–36. 10.3390/ma7020623. 10.3390/ma7020623 PMC5453079 28788479

[34] Wiesent L, Schultheiß U, Lulla P, Nonn A, Noster U. Mechanical properties of small structures built by selective laser melting 316 L stainless steel – a phenomenological approach to improve component design. Materialwissenschaft Werkst. 2020;51:1615–29. 10.1002/mawe.202000038.

[35] Li M, Yin T, Wang Y, Du F, Zou X, Gregersen H, et al. Study of biocompatibility of medical grade high nitrogen nickel-free austenitic stainless steel in vitro. Mater Sci Eng C Mater Biol Appl. 2014;43:641–8. 10.1016/j.msec.2014.06.038. 25175259 10.1016/j.msec.2014.06.038

[36] Wang HG, Yin TY, Ge SP, Zhang Q, Dong QL, Lei DX, et al. Biofunctionalization of titanium surface with multilayer films modified by heparin-VEGF-fibronectin complex to improve endothelial cell proliferation and blood compatibility. J Biomed Mater Res A. 2013;101:413–20. 10.1002/jbm.a.34339. 22865832 10.1002/jbm.a.34339

[37] Picone P, Sabatino MA, Ajovalasit A, Giacomazza D, Dispenza C, Di Carlo M. Biocompatibility, hemocompatibility and antimicrobial properties of xyloglucan-based hydrogel film for wound healing application. Int J Biol Macromol. 2019;121:784–95. 10.1016/j.ijbiomac.2018.10.078. 30342149 10.1016/j.ijbiomac.2018.10.078

[38] Goodman SL. Sheep, pig, and human platelet-material interactions with model cardiovascular biomaterials. J Biomed Mater Res. 1999;45:240–50. 10397982 10.1002/(sici)1097-4636(19990605)45:3<240::aid-jbm12>3.0.co;2-c

[39] Alomar Z, Concli F. A Review of the Selective Laser Melting Lattice Structures and Their Numerical Models. Adv Eng Mater 2020. 10.1002/adem.202000611.

[40] Amin Yavari S, Ahmadi SM, Wauthle R, Pouran B, Schrooten J, Weinans H, et al. Relationship between unit cell type and porosity and the fatigue behavior of selective laser melted meta-biomaterials. J Mech Behav Biomed Mater. 2015;43:91–100. 10.1016/j.jmbbm.2014.12.015. 25579495 10.1016/j.jmbbm.2014.12.015

[41] Demir AG, Previtali B. Additive manufacturing of cardiovascular CoCr stents by selective laser melting. Mater Des. 2017;119:338–50. 10.1016/j.matdes.2017.01.091.

[42] Fayazfar H, Salarian M, Rogalsky A, Sarker D, Russo P, Paserin V, et al. A critical review of powder-based additive manufacturing of ferrous alloys: Process parameters, microstructure and mechanical properties. Mater Des. 2018;144:98–128. 10.1016/j.matdes.2018.02.018.

[43] Bajaj P, Hariharan A, Kini A, Kürnsteiner P, Raabe D, Jägle EA. Steels in additive manufacturing: A review of their microstructure and properties. Mater Sci Eng: A. 2020;772:138633. 10.1016/j.msea.2019.138633.

[44] Zhang Y, Yang S, Zhao YF. Manufacturability analysis of metal laser-based powder bed fusion additive manufacturing—a survey. Int J Adv Manuf Technol. 2020;110:57–78. 10.1007/s00170-020-05825-6.

[45] Kurzynowski T, Gruber K, Stopyra W, Kuźnicka B, Chlebus E. Correlation between process parameters, microstructure and properties of 316 L stainless steel processed by selective laser melting. Mater Sci Eng: A. 2018;718:64–73. 10.1016/j.msea.2018.01.103.

[46] Yadollahi A, Shamsaei N, Thompson SM, Seely DW. Effects of process time interval and heat treatment on the mechanical and microstructural properties of direct laser deposited 316L stainless steel. Mater Sci Eng: A. 2015;644:171–83. 10.1016/j.msea.2015.07.056.

[47] Simson T, Emmel A, Dwars A, Böhm J. Residual stress measurements on AISI 316L samples manufactured by selective laser melting. Addit Manuf. 2017;17:183–9. 10.1016/j.addma.2017.07.007.

[48] Chen W, Voisin T, Zhang Y, Forien J-B, Spadaccini CM, McDowell DL, et al. Microscale residual stresses in additively manufactured stainless steel. Nat Commun. 2019;10:4338. 10.1038/s41467-019-12265-8. 31554787 10.1038/s41467-019-12265-8 PMC6761200

[49] Wang YM, Voisin T, McKeown JT, Ye J, Calta NP, Li Z, et al. Additively manufactured hierarchical stainless steels with high strength and ductility. Nat Mater. 2018;17:63–71. 10.1038/nmat5021. 29115290 10.1038/nmat5021

[50] Gorey TJ, Stull JA, Hackenberg RE, Clark CL, Hooks DE. Enhancing Surface Finish of Additively Manufactured 316L Stainless Steel with Pulse/Pulse Reverse Electropolishing. JOM. 2023;75:195–208. 10.1007/s11837-022-05558-9.

[51] Tyagi P, Goulet T, Riso C, Stephenson R, Chuenprateep N, Schlitzer J, et al. Reducing the roughness of internal surface of an additive manufacturing produced 316 steel component by chempolishing and electropolishing. Addit Manuf. 2019;25:32–8. 10.1016/j.addma.2018.11.001.

[52] Tyagi P, Brent D, Saunders T, Goulet T, Riso C, Klein K, et al. Roughness Reduction of Additively Manufactured Steel by Electropolishing. Int J Adv Manuf Technol. 2020;106:1337–44. 10.1007/s00170-019-04720-z.

[53] Hallab NJ, Bundy KJ, O’Connor K, Clark R, Moses RL. Cell adhesion to biomaterials: correlations between surface charge, surface roughness, adsorbed protein, and cell morphology. J Long Term Eff Med Implants. 1995;5:209–31. 10172729

[54] Habibzadeh S, Li L, Shum-Tim D, Davis EC, Omanovic S. Electrochemical polishing as a 316L stainless steel surface treatment method: Towards the improvement of biocompatibility. Corros Sci. 2014;87:89–100. 10.1016/j.corsci.2014.06.010.

[55] Bae I, Lim K-S, Park J-K, Song JH, Oh S-H, Kim J-W, et al. Evaluation of cellular response and drug delivery efficacy of nanoporous stainless steel material. Biomater Res. 2021;25:30. 10.1186/s40824-021-00232-8. 34565474 10.1186/s40824-021-00232-8 PMC8474832

[56] Pathirajah JP, Balamurugan S, Arvaj L, Weiss J, Barbut S. Influence of Different Stainless Steel Finishes on Biofilm Formation by Listeria monocytogenes. J Food Prot. 2022;85:1584–93. 10.4315/JFP-22-112. 36040237 10.4315/JFP-22-112

[57] Rodriguez A, Autio WR, McLandsborough LA. Effect of surface roughness and stainless steel finish on Listeria monocytogenes attachment and biofilm formation. J Food Prot. 2008;71:170–5. 10.4315/0362-028X-71.1.170. 18236679 10.4315/0362-028x-71.1.170

[58] Hilbert LR, Bagge-Ravn D, Kold J, Gram L. Influence of surface roughness of stainless steel on microbial adhesion and corrosion resistance. Int Biodeterior Biodegrad. 2003;52:175–85. 10.1016/S0964-8305(03)00104-5.

[59] Huang Q, Yang Y, Hu R, Lin C, Sun L, Vogler EA. Reduced platelet adhesion and improved corrosion resistance of superhydrophobic TiO₂-nanotube-coated 316L stainless steel. Colloids Surf B Biointerfaces. 2015;125:134–41. 10.1016/j.colsurfb.2014.11.028. 25481855 10.1016/j.colsurfb.2014.11.028

[60] Shang Y, Yuan Y, Li D, Li Y, Chen J. Effects of scanning speed on in vitro biocompatibility of 316L stainless steel parts elaborated by selective laser melting. Int J Adv Manuf Technol. 2017;92:4379–85. 10.1007/s00170-017-0525-5.

[61] Yang Z, Wang J, Luo R, Maitz MF, Jing F, Sun H, et al. The covalent immobilization of heparin to pulsed-plasma polymeric allylamine films on 316L stainless steel and the resulting effects on hemocompatibility. Biomaterials. 2010;31:2072–83. 10.1016/j.biomaterials.2009.11.091. 20022107 10.1016/j.biomaterials.2009.11.091

[62] Gorbet MB, Sefton MV. Biomaterial-associated thrombosis: roles of coagulation factors, complement, platelets and leukocytes. Biomaterials. 2004;25:5681–703. 10.1016/j.biomaterials.2004.01.023. 15147815 10.1016/j.biomaterials.2004.01.023

[63] Ollivier V, Roques C, Receveur N, Gratz M, Feldman L, Letourneur D, et al. Bioreactivity of stent material: Activation of platelets, coagulation, leukocytes and endothelial cell dysfunction in vitro. Platelets. 2017;28:529–39. 10.1080/09537104.2016.1252836. 28032527 10.1080/09537104.2016.1252836

[64] Wood MH, Payagalage CG, Geue T. Bovine Serum Albumin and Fibrinogen Adsorption at the 316L Stainless Steel/Aqueous Interface. J Phys Chem B. 2018;122:5057–65. 10.1021/acs.jpcb.8b01347. 29709171 10.1021/acs.jpcb.8b01347

[65] Eaton JW, Keogh JR. Albumin binding surfaces for biomaterials. J Lab Clin Med. 1994;124:537–45. 7523554

[66] Kottke-Marchant K, Anderson JM, Umemura Y, Marchant RE. Effect of albumin coating on the in vitro blood compatibility of Dacron arterial prostheses. Biomaterials. 1989;10:147–55. 10.1016/0142-9612(89)90017-3. 2524222 10.1016/0142-9612(89)90017-3

[67] Brevig T, Holst B, Ademovic Z, Rozlosnik N, Røhrmann JH, Larsen NB, et al. The recognition of adsorbed and denatured proteins of different topographies by beta2 integrins and effects on leukocyte adhesion and activation. Biomaterials. 2005;26:3039–53. 10.1016/j.biomaterials.2004.09.006. 15603799 10.1016/j.biomaterials.2004.09.006

[68] Werthén M, Sellborn A, Källtorp M, Elwing HB, Thomsen P. In vitro study of monocyte viability during the initial adhesion to albumin- and fibrinogen-coated surfaces. Biomaterials. 2001;22:827–32. 10.1016/S0142-9612(00)00246-5. 11246951 10.1016/s0142-9612(00)00246-5

[69] Jaffer IH, Fredenburgh JC, Hirsh J, Weitz JI. Medical device-induced thrombosis: what causes it and how can we prevent it? J Thromb Haemost. 2015;13:S72–81. 10.1111/jth.12961. 26149053 10.1111/jth.12961

[70] Hansi C, Arab A, Rzany A, Ahrens I, Bode C, Hehrlein C. Differences of platelet adhesion and thrombus activation on amorphous silicon carbide, magnesium alloy, stainless steel, and cobalt chromium stent surfaces. Catheter Cardiovasc Inter. 2009;73:488–96. 10.1002/ccd.21834. 10.1002/ccd.21834 19235237

[71] Hietala E-M, Maasilta P, Juuti H, Nuutinen J-P, Harjula ALJ, Salminen U-S, et al. Platelet deposition on stainless steel, spiral, and braided polylactide stents. A comparative study. Thromb Haemost. 2004;92:1394–401. 10.1160/TH04-02-0124. 15583749 10.1160/TH04-02-0124

[72] Ferrara LA, Fleischman AJ, Togawa D, Bauer TW, Benzel EC, Roy S. An in vivo Biocompatibility Assessment of MEMS Materials for Spinal Fusion Monitoring. Biomed Microdevices. 2003;5:297–302. 10.1023/A:1027305729456.

[73] Muthusubramaniam L, Lowe R, Fissell WH, Li L, Marchant RE, Desai TA, et al. Hemocompatibility of silicon-based substrates for biomedical implant applications. Ann Biomed Eng. 2011;39:1296–305. 10.1007/s10439-011-0256-y. 21287275 10.1007/s10439-011-0256-y PMC3069312

[74] Michelson AD, Barnard MR, Hechtman HB, MacGregor H, Connolly RJ, Loscalzo J, et al. In vivo tracking of platelets: circulating degranulated platelets rapidly lose surface P-selectin but continue to circulate and function. Proc Natl Acad Sci USA. 1996;93:11877–82. 10.1073/pnas.93.21.11877. 8876231 10.1073/pnas.93.21.11877 PMC38152

[75] Brambilla M, Josefsson EC, Ramstrom S, Di Minno A, Di Minno MND, Gangatirkar P, et al. Biomarkers of in vivo platelet activation in coronary artery disease: a systematic review and meta-analysis: communication from the SSC of the ISTH. J Thromb Haemost. 2025;23:3989–4009. 10.1016/j.jtha.2025.07.014. 40685139 10.1016/j.jtha.2025.07.014

[76] Patel M, Parrish A, Serna C 3rd, Jamiolkowski M, Srinivasan K, et al. Molecular Biomarkers for In Vitro Thrombogenicity Assessment of Medical Device Materials. J Biomed Mater Res B Appl Biomater. 2024;112:e35491. 10.1002/jbm.b.35491. 39340365 10.1002/jbm.b.35491 PMC11661467

[77] Gemmell CH. Activation of platelets by in vitro whole blood contact with materials: increases in microparticle, procoagulant activity, and soluble P-selectin blood levels. J Biomater Sci Polym Ed. 2001;12:933–43. 10.1163/156856201753113114. 11718486 10.1163/156856201753113114

[78] Li X-M, Li H-Z, Wang S-P, Huang H-M, Huang H-H, Ai H-J, et al. MRI-compatible Nb-60Ta-2Zr alloy used for vascular stents: haemocompatibility and its correlation with protein adsorption. Mater Sci Eng C Mater Biol Appl. 2014;42:385–95. 10.1016/j.msec.2014.05.051. 25063132 10.1016/j.msec.2014.05.051

[79] Weber M, Steinle H, Golombek S, Hann L, Schlensak C, Wendel HP, et al. Blood-Contacting Biomaterials: In Vitro Evaluation of the Hemocompatibility. Front Bioeng Biotechnol. 2018;6:99 10.3389/fbioe.2018.00099. 30062094 10.3389/fbioe.2018.00099 PMC6054932

[80] Waterhouse A, Yin Y, Wise SG, Bax DV, McKenzie DR, Bilek MM, et al. The immobilization of recombinant human tropoelastin on metals using a plasma-activated coating to improve the biocompatibility of coronary stents. Biomaterials. 2010;31:8332–40. 10.1016/j.biomaterials.2010.07.062. 20708259 10.1016/j.biomaterials.2010.07.062

