# Supplementary material for: Hemocompatibility and cytotoxicity evaluation of additively manufactured and surface-treated 316 L stainless steel aortic stents using laser powder bed fusion (L-PBF)
Source: J Mater Sci Mater Med. 2026 May 27;37(1):61. doi: 10.1007/s10856-026-07073-8 (PMC13216169; doi:10.1007/s10856-026-07073-8)
Supplement: Supplementary file 1 — Supplementary information [file 10856_2026_7073_MOESM1_ESM.docx]

**Supplementary Material and Methods.**

Supplementary Table S1: Chemical composition of the 316L powder used in this study

| **Element** | **Fe** | **Si** | **Mn** | **P** | **Cr** | **Mo** | **Ni** | **C** | **S** | **N** |
| --- | --- | --- | --- | --- | --- | --- | --- | --- | --- | --- |
| wt-% | Balance | 0,26 | 1,5 | <0,01 | 16,6 | 2,35 | 11,4 | 0,02 | 0,003 | 0,07 |
| Specification | Balance | ≤1.00 | ≤2.00 | ≤0.045 | 16.50-18.50 | 2.00-2.50 | 10.00-13.00 | ≤3.00 | ≤0.015 | ≤1.00 |

Supplementary Table S2: List of all used antibodies for immunofluorescence (IF) and flow cytometric analysis (FACS)

| **Antibody** | **manufacturer** | **article no** | **dilution** | **method** |
| --- | --- | --- | --- | --- |
| Monoclonal rabbit anti-human CD61 (GPIIIa, platelet marker) | Invitrogen, Waltham, MA, USA | MA5-32077 | 1:200 | IF |
| Monoclonal mouse anti-human CD62P (P-selectin) | Santa Cruz, Heidelberg, Germany | SC-8419 | 1:200 | IF |
| Polyclonal rabbit anti-human MPO (myeloperoxidase) | Dako, Santa Clara, CA, USA | A0398 | 1:1200 | IF |
| Monoclonal mouse anti-human CD11b (leukocytic activation marker) | Biozol, Eching, Germany | MBS245100 | 1:200 | IF |
| Monoclonal rabbit anti-human CD62L (L-selectin) | Abcam, Cambridge, UK | Ab264045 | 1:100 | IF |
| Polyclonal donkey anti-rabbit IgG-AlexaFluor488 | Dianova, Hamburg, Germany | 711-545-152 | 1:300 | IF |
| Polyclonal donkey anti-rabbit IgG-AlexaFluor594 | Dianova, Hamburg, Germany | 711-585-152 | 1:300 | IF |
| Polyclonal donkey anti-mouse IgG-AlexaFluor594 | Dianova, Hamburg, Germany | 715-585-150 | 1:300 | IF |
| Monoclonal mouse anti-human CD61-PerCPCy5.5 | BD Pharmingen, Heidelberg, Germany | 564173 | 5 µL | FACS |
| Monoclonal mouse anti-human PAC1-FITC (GPIIb/IIIa, Fibrinogen receptor) | BD Biosciences, San Jose, CA, USA | 340507 | 20 µL | FACS |
| Monoclonal mouse anti-human CD62P-PE | BD Biosciences, San Jose, CA, USA | 348107 | 20 µL | FACS |
| Monoclonal mouse anti-human CD62L-FITC | BioLegend, San Diego, CA, USA | 304804 | 5 µL | FACS |
| Monoclonal mouse anti-human CD11b-PE | BioLegend, San Diego, CA, USA | 301306 | 5 µL | FACS |
| Monoclonal mouse anti-human CD62L-PerCPCy5.5 | BioLegend, San Diego, CA, USA | 304824 | 5 µL | FACS |
| Monoclonal mouse anti-human CD14-FITC (monocyte marker) | BD Pharmingen, Heidelberg, Germany | 555397 | 20 µL | FACS |
| Monoclonal mouse anti-human CD45-APC (common leukocyte marker) | BD Pharmingen, Heidelberg, Germany | 555485 | 20 µL | FACS |

FITC, fluorescein isothiocyanate; PE, phycoerythrin; APC, allophycocyanin; PerCPCy5.5, peridinin chlorophyll protein-Cyanine 5.5;

Protocols with fluorophore-conjugated materials or antibodies were always processed in the dark. Used antibodies were listed in Supplementary Table 2.

**Details of used materials**

Fluorophore-conjugated materials including fibrinogen-AF488 (AlexaFluor; 10 mg/mL; Thermo Fisher, Waltham, MA, US); SytoxGreen (SG; 5 mM in DMSO; Thermo Fisher Scientific) and Rhodamine-Phalloidin (RP; 6.6 µM; Invitrogen) were used.

Normal donkey serum (NDS) and bovine serum albumin (BSA) were from Roth (Karlsruhe, Germany). Coldwater Fish Gelatine; TritonX100; Tween20; PLL (Poly-L-Lysine, 0.1 %, P8920); PMA (Phorbol Myristate Acetate, 1 mM in dimethylformamid); fMLP (N-formylmethionyl-leucyl-phenylalanine, 1 mM in dimethylformamid); Trypsin/EDTA (10x); Dulbecco´s modified Eagle´s Medium (DMEM, low glucose); FBS (fetal bovine serum, 10%); amphotericin; gentamycin and L-glutamine (2 mM, G7513); AB-serum were from Sigma-Aldrich (Steinheim, Germany). TNF (Tumor Necrosis Factor, 1 µg/mL in PBS/0.1%BSA); Fluoromount-G (#00-4958) and Fluoromount-G-DAPI (#00-4959) mounting medium were from Thermo Fisher Scientific (Waltham, MA, USA).

Different buffers were used: TBS (Tris buffered saline, 0.42 g Tris base, 2.6 g TrisHCl, 8.8 g NaCl ad 1 L H_2_O, pH7.4); TBST0.1% (TBS + 0.5 % TritonX100 + 0.5 % Tween20); TBST0.05% and TBST0.005% (1:2 and 1:20 dilution of TBST0.1% with TBS); blocking buffer (TBS + 1 % BSA + 2 % NDS + 0.2 % Coldwater Fish Gelatine + 0.05 % TritonX100 + 0.05 % Tween20); HTP (Tyrode´s Sol., Hepes-buffered, J67607, Thermo Fisher Scientific; containing 2 mM Mg^2+^/Ca^2+^) pH 7.4; HTP/0.5%BSA (HTP + 0.5 % BSA); PBS/0.5%BSA (PBS (phosphate buffered saline) + 0.5 % BSA). Culture medium consisted of DMEM supplemented with FBS (10%), amphotericin, gentamycin and L-glutamine.

The centrifuge 5810 (Eppendorf, Hamburg, Germany) was used throughout the study.

**Test material for cytotoxicity evaluation**

The as-build (AB) as well as different post-processed samples were tested for cytotoxicity. Post-processing treatment included combinations of CE (chemically etched), EP (electropolished), HT, heat-treated, ME (mechanically expanded). The final test sample (TS) was defined as AB+CE+EP+HT+ME.

**Image analysis of adherent platelets on TS after Rhodamine-Phalloidin staining (RP)**

Twenty randomly selected locations along the TS (Figure 8A) were photographed in the red channel (16x magnification). NIH ImageJ software (Bethesda, MD, USA) was used for image processing and analysis of the surface coverage and number of particles per mm^2^. Variability in background staining was minimized with post-image thresholding. All image analysis were performed by an investigator blinded to the production patch to avoid the introduction of bias. First, the scale bare (fixed on each image) was adjusted to allow the determination of surface areas in mm^2^. Due to the variable shape of the TS sections, an individual region of interest (ROI) was defined for each image. ROIs of the struts (NO, SO, SW, NW) ranged between 0.36 and 0.96 mm^2^, while the corners (N, O, S, W) allowed ROI definitions between 0.76 and 2.39 mm^2^. The “Analysis Particles” function was used to determine the number and area of the particles within the ROIs. The cellular coverage was defined as the area of all particles relative to the total ROI of the individual image (%). Individual particle areas were subdivided into three ranges (<10, 10-100, >100 µm^2^) to demonstrate single platelets, small and large platelet aggregates. This classification based on the size of normal platelets with a maximum diameter of 3 µm (area, 7.1 µm^2^). The subdivision was determined relative to the total amount of particles per ROI.

**Figure Legends of Supplementary Figures**

Supplementary Figure S 1:

Flow cytometric analysis of isolated platelets to visualize the expression of PAC-1-FITC and CD62P-PE (A, B) and quantify the extent of Fibrinogen-AF488-binding activity (C, D). (A) Platelets were identified according to their forward (FSC) and low SSC signal. (B) These cells were divided into PAC1-FITC-positive and/or CD62P-PE-positive (or both) platelet populations (dotplot). The proportion of positive cells as well as its median fluorescence intensity (median FI) (in the respective quadrant) was analyzed. (C) Platelets were identified by their CD61-positive fluorescence and 90 degree light scatter (SSC). This population was subdivided into non-stimulated platelets (Fibrinogen-AF488-negative) or ADP-stimulated platelets (Fibrinogen-AF488-positive).
