# Supplementary figures and images for: Hemocompatibility and cytotoxicity evaluation of additively manufactured and surface-treated 316 L stainless steel aortic stents using laser powder bed fusion (L-PBF)
Source: J Mater Sci Mater Med. 2026 May 27;37(1):61. doi: 10.1007/s10856-026-07073-8 (PMC13216169; doi:10.1007/s10856-026-07073-8)

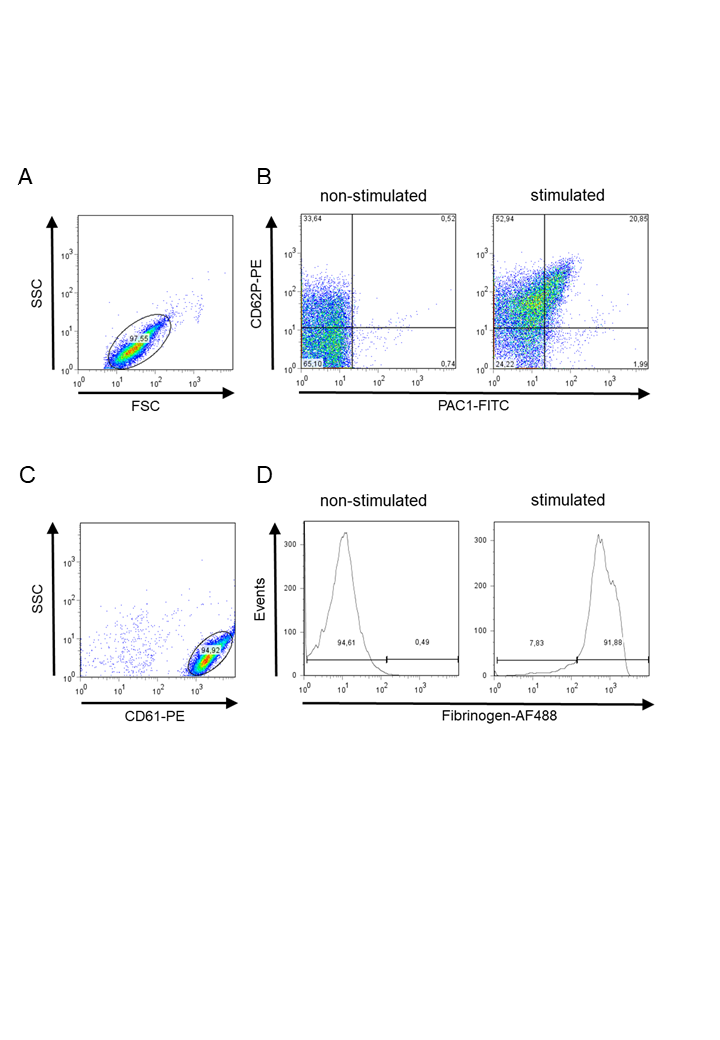

Supplement: Supplementary file 2 — Supplementary information [file 10856_2026_7073_MOESM2_ESM.tif]
